# Impact of analytic decisions on test–retest reliability of individual and group estimates in functional magnetic resonance imaging: A multiverse analysis using the monetary incentive delay task

**DOI:** 10.1162/imag_a_00262

**Published:** 2024-09-10

**Authors:** Michael I. Demidenko, Jeanette A. Mumford, Russell A. Poldrack

**Affiliations:** Department of Psychology, Stanford University, Stanford, CA, United States

**Keywords:** test–rest reliability, intraclass correlation, Jaccard similarity, functional magnetic resonance imaging, monetary incentive delay task, individual differences

## Abstract

Empirical studies reporting low test–retest reliability of individualblood oxygen-level dependent (BOLD) signal estimates in functional magneticresonance imaging (fMRI) data have resurrected interest among cognitiveneuroscientists in methods that may improve reliability in fMRI. Over the lastdecade, several individual studies have reported that modeling decisions, suchas smoothing, motion correction, and contrast selection, may improve estimatesof test–retest reliability of BOLD signal estimates. However, it remainsan empirical question whether certain analytic decisions*consistently*improve individual- and group-levelreliability estimates in an fMRI task across multiple large, independentsamples. This study used three independent samples (*N*s: 60, 81,119) that collected the same task (Monetary Incentive Delay task) across tworuns and two sessions to evaluate the effects of analytic decisions on theindividual (intraclass correlation coefficient [ICC(3,1)]) and group(Jaccard/Spearman*rho*) reliability estimates of BOLD activityof task fMRI data. The analytic decisions in this study vary across fourcategories: smoothing kernel (five options), motion correction (four options),task parameterizing (three options), and task contrasts (four options), totaling240 different pipeline permutations. Across all 240 pipelines, the median ICCestimates are consistently low, with a maximum median ICC estimate of .43– .55 across the 3 samples. The analytic decisions with the greatestimpact on the median ICC and group similarity estimates are the*ImplicitBaseline*contrast, Cue Model parameterization, and a largersmoothing kernel. Using an*Implicit Baseline*in a contrastcondition meaningfully increased group similarity and ICC estimates as comparedwith using the*Neutral*cue. This effect was largest for the CueModel parameterization; however, improvements in reliability came at the cost ofinterpretability. This study illustrates that estimates of reliability in theMID task are consistently low and variable at small samples, and a highertest–retest reliability may not always improve interpretability of theestimated BOLD signal.

## 1 Introduction

Reliability in functional magnetic resonance imaging (fMRI) is essential toindividual differences research as well as for the development of clinicalbiomarkers. Unfortunately, numerous studies have demonstrated that reliability ofindividual estimates in fMRI is low (Elliott et al.,2020;Noble et al., 2019) and thereliability of group estimates in statistical maps is sensitive to varyinganalytical decisions made by researchers (Botvinik-Nezer et al., 2020).^*^Poor reliability can hamper validity in cognitiveneuroscience research, reducing the ability to uncover brain-behavior effects (Hedge et al., 2018;Nikolaidis et al., 2022) and the ability to detectdifferences in distinct brain states and individual traits (Gell et al., 2023;Kragelet al., 2021). It remains to be seen whether certain analytic decisions*consistently*reduce individual and/or group reliabilityestimates of blood oxygen-level dependent (BOLD) activity across measurementoccasions in univariate task fMRI analyses.

FMRI analysis involves a range of analytic decisions (Caballero-Gaudes & Reynolds, 2017;Soares et al., 2016) that can result in a vast number ofstatistical brain maps across which BOLD activity can vary subtly or substantially(Bowring et al., 2022;Carp, 2012;Li et al.,2021). Simple decisions, such as using different MNI template brains, cangreatly affect the agreement between parameter estimates between two preprocessingpipelines (Li et al., 2021). Furthermore, theapproach used to model a task design can also alter interpretations (Botvinik-Nezer et al., 2020). As a result of numerousarbitrary choices, preprocessing and task modeling decisions can significantlyimpact the reliability of voxel/region of interest (ROI) estimates (Dubois & Adolphs, 2016).

Different metrics of reliability provide quantitative indices of the consistency (orsimilarity) of estimates of BOLD activity in specific brain regions (or voxels)during fMRI task activation across repeated measurement occasions (Bennett & Miller, 2013). Researchers can quantifythe consistency of two repeated measures in terms of estimated effects (continuous)and/or the presence/absence of a significant effect (binary). In terms of thecontinuous effects, reliability is an estimate of the consistency of the numericalrepresentation of a measure (e.g., BOLD activity in the supplementary motor areaduring a finger tapping task;Witt et al.,2008) of a mental process (e.g., index finger movement) across repeatedmeasurement occasions within an*individual*(e.g., task fMRIcontrasts across two or more sessions, which can be hours, days, or weeks apart).This form of reliability is usually calculated using an intraclass correlation (ICC)at the whole brain (i.e., voxel-wise) and/or ROI level. In terms of binary estimatesof an effect, reliability is an estimate of an experimental task’s (e.g.,finger tapping task;Witt et al., 2008)ability to evoke statistically significant activation (above a prespecifiedthreshold) in the same regions for*groups*of subjects for aspecific condition (e.g., finger movement vs. rest) across measurement occasions(e.g., task fMRI contrasts across two or more scanning sessions). Binary estimatesof reliability are often calculated using Dice (Rombouts et al., 1998) or Jaccard’s similarity coefficients(Maitra, 2010). Together, these two formsof reliability reflect the consistency (or agreement) in either the magnitude or thebinary statistical significance of an experimental effect occurring during taskfMRI.

Traditionally, empirical studies have referred to the “robustness” ofabove-threshold activation signals in group fMRI analyses as an implicit indicatorof reliability of an fMRI task. While a useful heuristic,Fröhner et al. (2019)argued that robustnessacross measurement occasions only represents reliability of*group*(overall average) BOLD activity and does not accurately represent*individual*variability in BOLD activity. In addition,thresholding is a nonlinear operation that can result in substantial variability(Cohen & DuBois, 1999). Whenquantifying reliability of BOLD activity in the brain, researchers often report anICC or a similarity coefficient for task fMRI (Bennett & Miller, 2013;Fröhner et al., 2019). The lack of standardization makes itchallenging to precisely quantify reliability, relative to individual differences,and assess the impact of different fMRI analysis decisions on continuous and binaryestimates of reliability.

To date, several studies have examined the impact of analytic decisions, such asspatial smoothing, motion correction, and contrast modeling, on individual estimatesof reliability of task fMRI.Caceres et al.(2009;*n*= 10) found that an optimal smoothingkernel size of 8–10 FWHM (full-width half-maximum) on a 1.5T scanner with3.75 mm voxels improved reliability. Results regarding the impact of motioncorrection on reliability are mixed, withGorgolewski et al. (2013;*n*= 11) reporting apositive effect on reliability, whilePlichta et al.(2012;*n*= 25) reporting no effect during areward task and a negative effect during a faces and N-back task on reliability.However, in a large, young sample,Kennedy et al.(2022;*n*= 5,979 – 6,593) reported thatexcluding high motion subjects modestly improved reliability. Finally,Han et al. (2022;*n*=29 – 120) andKennedy et al. (2022;*n*= 5,979 – 6,593) reported that using animplicit baseline for different tasks (e.g., rest phase during the task) rather thana neutral cue increased reliability across measurement occasions. Some, but not all,of these findings are consistent with a previous review of the fMRI reliabilityliterature (Bennett & Miller, 2013),which suggests that motion, spatial smoothing, and task signal likely impactreliability in task fMRI. However, differences in modeling decisions across thesestudies leave an important question unanswered: Are there certain analytic decisionsthat*consistently*improve reliability (e.g., ICC) of neuralactivity for an fMRI task across samples?

The ICC is a statistic adopted from behavioral research to estimate reliability ofobserved scores across measurement occasions (Bartko,1966;Fisher, 1934;Shrout & Fleiss, 1979;Spearman, 1904). In the context of multisession data,there are several ways to estimate an ICC, but for typical univariate fMRI studies,two specific types (ICC[2,1] and ICC[3,1]) are recommended (For a discussion, seeNoble et al., 2021). As describedelsewhere (Bennett & Miller, 2013;Fisher, 1934), the ICC is similar to theproduct moment correlation. Unlike the product moment correlation, which estimatesseparate means and variances between distinct classes (e.g., age and height), theICC estimates the mean and variances within a single class (e.g., measure). For twoor more variables from a single class, test–retest reliability estimates theconsistency (or agreement) of the observed scores across the measurement occasions.Using the correlation coefficient as an example, if there are no differences insubjects’ scores across two measurement occasions, the correlationcoefficient would be 1.0. However, if the measure is affected by systematic and/orunsystematic error across measurement occasions, this would impact the covariancebetween observed scores across subjects and decrease the linear association betweenmeasures across the two occasions. Unlike the product moment correlation, however,the ICC factors out measurement bias which reflects the reproducibility of observedscores across measurement occasions (Liu et al.,2016). While the correlation between two occasions (**A**= [1, 3, 6, 9, 12] &**B**= 3 x**A**= [3, 9, 18, 27, 36]) may be perfect (*r*_AB_= 1.0), the consistency in observed scores between the two measurementoccasions would be lower (ICC[3,1] = .60). In fMRI, the reliability of theBOLD signal may be impacted by biological (e.g., differences in BOLD across brainregion), analytic (e.g., task design and analytic decisions), and participant-levelfactors (e.g., practice effects, motion, habituation, and/or development). Thesefluctuations, whether typical or atypical, may contribute to observed differencesand the reduced consistency in scores across measurement occasions, leading todecreased estimates of reliability.

As discussed in prior work on fMRI reliability (Bennett & Miller, 2010,^2013^;Caceres et al., 2009;Chen et al., 2017;Herting et al., 2017;Noble et al., 2021), the ICC decomposes the total variance of the dataacross all subjects and sessions into two key parts:*Between-subject*and*Within-subject*variance(for statistical formulas and discussion of ICC, seeLiljequist et al. (2019)and flowchart inMcGraw & Wong (1996, p. 40)). The ICC estimate canbe altered by increasing the differences in BOLD activity between subjects (e.g.,subjects differ more in BOLD activity in index finger movements) and/or ensured thatBOLD activity within subjects is more similar across scans (e.g., BOLD activity inresponse to finger movements vs. rest for Subject A is consistent across Session 1and Session 2). Some have argued that the low*between-subject*variability may be a reason for low reliability of behavioral responses inexperimental tasks that are commonly used in fMRI (Hedge et al., 2018). However, there is little empirical research onwhether the culprit in the reportedly low reliability of fMRI signal acrossmeasurement occasions is a*decreased between-subject*and/or an*increased within-subject*variability. It also remains an openquestion whether certain analytic decisions differentially impact thebetween-/within-subject variance and consistently improve reliability acrossdifferent samples with the same task. As it relates to prediction and globalsignal-to-noise ratio, evidence fromChurchill etal. (2015;*n*= 25) suggests that there are likelyto be optimal preprocessing pipelines; however, the degree to which these differacross datasets and individuals is currently unknown.

The current study uses a multiverse (Steegen et al.,2016) of analytic alternatives to simultaneously evaluate the effects ofanalytic decisions on the continuous and binary reliability estimates of neuralactivity in task fMRI in three samples. The three samples are administered with thecomparable Monetary Incentive Delay (MID) task during fMRI across two runs and twosessions. The purpose of multiple samples with the same task design is to evaluatethe consistency in findings across studies that vary in their sample populations buthave overlapping task designs, as little evidence exists on the*consistency*of reliability estimates for the same task acrossindependent samples.**Aim 1**evaluates the effects of analytic decisionsincluding task model smoothing, motion correction, parameterization (i.e.,modeling), and task contrasts on the impacts on reliability, calculated usingICC(3,1) for individual [continuous] beta estimates and Jaccard’s similaritycoefficient using significance thresholded group [binary] estimates(*p*< .001, uncorrected) and Spearman correlation group[continuous] estimates. The decisions are noted inTable 1.**Aim 1 Hypothesis**is that the highest produced ICCand similarity coefficient/correlation is for the model decisions indicated by**bold**for A–D categories inTable 1. This, in part, is because the analytic strategy includes (1)motion correction techniques that limit the number of noisy (high motion) subjectsand reduce the number of degrees of freedom that are lost due to censoring, (2) anoptimal smoothing for the size of voxels, and (3) the highest activation contrastfrom a task modeling phase that is relatively efficient. We hypothesize this to bemore so the case for the older (e.g., AHRB/MLS) than younger samples (e.g., ABCD)due to changes occurring as a result of development (Herting et al., 2017;Noble et al.,2021). Due to the lack of information regarding how the between-subjectvariance (BS) and within-subject variance (WS) are impacted by analytic choices intask fMRI analyses,**Aim 2**evaluates the change in BS and WS components.Due to the poor reliability of individual estimates in task fMRI (Elliott et al., 2020), reported evidence of highbetween-subject variability in BOLD activity (Turneret al., 2018), and limited evidence on changes in BS and WS variancecomponents in the MID task, we do not have a specific**Aim 2 Hypothesis**.Finally, seeing as the ICC is, in some ways, similar to a moment product correlation(Bennett & Miller, 2010) whichstabilizes at larger sample sizes (Grady et al.,2021;Marek et al., 2022;Schönbrodt & Perugini, 2013),**Aim 3**evaluates at what sample the ICC stabilizes using the mostoptimal pipeline (e.g., highest median ICC) used in Aim 2. Stability of Jaccardcoefficient group maps is not considered in Aim 3 as these estimates are sensitiveto significant thresholding. Using the evidence from prior work on correlations(Grady et al., 2021;Schönbrodt & Perugini, 2013), the**Aim3 Hypothesis**is that the ICC will stabilize at a sample size between 150and 500.

**Table 1.** Proposed analytic permutations: 360 total modeling combinations for MIDtask.

First-level pipeline decisions	Options
A. Smoothing (FWHM)	
1. 1.5x voxel	ON / OFF
2. 2x voxel	ON / OFF
**3. 2.5x voxel**	ON / OFF
4. 3x voxel	ON / OFF
5. 3.5x voxel	ON / OFF
B. Motion Correction	
1. None	ON / OFF
2. Regress: Translation/Rotation (x,y,z) + Derivative (x,y,z)	ON / OFF
3. Regress: Regress: Translation/Rotation (x,y,z) + Derivative (x,y,z) + First 8 aCompCor Components	ON / OFF
4. Regress: Translation/Rotation (x,y,z) + Derivative (x,y,z) + First 8 aCompCor Components + Censor High Motion Volumes (FD ≥ .9)	ON / OFF
^#^ **5. Regress: Translation/Rotation (x,y,z) + Derivative (x,y,z) + First 8 aCompCor Components, Exclude mean FD ≥ .9**	ON / OFF
^#^ 6. Regress: Translation/Rotation (x,y,z) + Derivative (x,y,z) + First 8 aCompCor Components + Censor High Motion Volumes, Exclude mean FD ≥ .9	ON / OFF
C. Task Modeling	
1. MID: Cue Onset, Cue Duration only	ON / OFF
**2. MID: Cue Onset, Cue + Fixation Duration**	ON / OFF
3. MID: Fixation onset, Fixation Duration	ON / OFF
D. Task Contrasts	
1. MID: Big Win > Neutral	ON / OFF
**2. MID: Big Win > Implicit**	ON / OFF
3. MID: Small Win > Neutral	ON / OFF
4. MID: Small Win > Implicit	ON / OFF

**Bold**: Model hypothesized to produce the highesttest–retest reliability; aCompCor: Anatomical Component BasedNoise Correction; MID: Monetary Incentive Delay task; FD: Frame-wisedisplacement.

# Due to the lack of low motion subjects (zero mean FD < .90 in 2/3samples), this decision was not included in the Stage 2 analyses,resulting in 240 analytic models.

## 2 Methods

To answer the questions proposed in Aim 1 and Aim 2, this study will require multiplesamples and tasks to obtain a comprehensive view of how analytic decisions impactgroup and individual reliability metrics (Aim 1) and how BS and WS are impacted (Aim2) across multiple samples and similar MID task. We use three samples with subjectsthat have at least two repeated sessions of data. To answer the question about thesample at which ICC stabilizes (Aim 3), we use the repeated session data from alarge consortium sample.

The studies were selected based on two criteria. First, the goal is to derive groupand individual estimates of reliability using sample sizes that are larger than thereported median sample size in fMRI research. The median reported sample size infMRI is <30 subjects (Poldrack et al.,2017;Szucs & Ioannidis,2017). From the review of task fMRI reliability byBennett and Miller (2010), the median sample forindividual (continuous) reliability is 10 subjects (mean = 10.5 [range= 1 to 26]) and that for group (binary) reliability is 9.5 subjects (mean= 11.2 [range = 4 to 45]). Recent reviews and analyses of task fMRIreliability suggest that sample sizes are increasing, but they remain below themedian sample size commonly observed in task fMRI studies. Specifically, inElliott et al. (2020), the median sample sizefor individual reliability in their meta-analysis is 18 subjects (mean =26.4, range = 5 to 467) and their independent analyses consisted of sampleswith 45 and 20 subjects, respectively. Second, the goal is to limit the interactionbetween reliability estimates and unknown features of the data, such as the mentalprocesses, to get a sense of how the analytic pipeline impacts reliability estimates*consistently*across a similar task design. Thus, the 3 samplesdescribed below exceed N > 50 and use a nearly identical task that is knownto evoke a strong BOLD response in specific reward-related brain regions to achievethese two goals.

### 2.1 Participants ^†^

#### 2.1.1 Adolescent Brain Cognitive Development (ABCD) study

The ABCD Study^®^is a longitudinal national study that wasdesigned to study the change in behavioral and biological measurementsacross development (Volkow et al.,2018). Informed consent was obtained from all participants forbeing included in the study. The focus here is on the 4.0 brain imaging datathat are released by the ABCD-BIDS Community Collection (ABCC;Feczko et al., 2021). As of February2024, the ABCC data contain year 1 (approximately 11,000, participants aged9–10 years) and year 2 (approximately 7,000 participants, age11–13 years) fMRI data. For Aims 1 and 2, we use a subsample of ABCDparticipants at the University of Michigan site (site = 13) withmaximum clean data available as this would be sufficient to test thehypotheses and limit site and scanner effects. For Aim 3, we use a subsampleof N = 2,000 of the maximum clean data available from the ABCC sampleand use an adaptive design to answer at which*N*ICCstabilizes. To reduce the use of unnecessary computational resources, theanalyses are first performed in N = 525. If the difference betweenaverage ICC estimate for interval N*_i_*andN*_i -1_*is >.15, the sample will beextended to*N*= 1,000, adding*N*= 500, until the plotted estimates are stable. As described elsewhere(Casey et al., 2018), the studycollected fMRI data during the Stopsignal, Emotional N-back, and MID tasks.Reliability of consortium-derived region of interest level data for year 1and year 2 has been reported elsewhere (Kennedy et al., 2022). We expand on these findings by evaluatinghow consistent these results are across studies and how analytic decisionsimpact estimates of reliability. Here, we use the raw BOLD timeseries fromthe MID task as this is consistent with the two other studies describedbelow.

##### 2.1.2 Michigan Longitudinal Study (MLS)

The MLS is a longitudinal study focused on the change in behavioral andbiological measurements across development. Informed consent wasobtained from all participants for being included in the study. Asdescribed elsewhere (Martz et al.,2016;Zucker et al.,2000), the MLS includes the Neuropsychological Risk cohort.The MLS Neuropsychological Risk cohort contains year 1 (approximately159 participants, age 18–24 years) and year 2 (approximately 150participants, age 20–26 years) fMRI data. The study collectedfMRI data during the affective word and MID tasks. Here, we use the rawBOLD data from the MID task as it is consistent with the ABCD study andAdolescent Risk Behavior Study (described below).

##### 2.1.3 Adolescent Risk Behavior (AHRB) study

The AHRB study is a longitudinal study focused on the change inbehavioral and biological measurements across development. Informedconsent was obtained from all participants for being included in thestudy. The AHRB study contains year 1 (approximately 108 participants,age 17–20 years) and year 2 (approximately 66 participants, age19–22 years). The study collected fMRI data during the EmotionalFaces and MID tasks. Here, we use the raw BOLD data from the MID task asit is consistent with the MLS and ABCD study.

### 2.2 FMRI task, data, and preprocessing

#### 2.2.1 FMRI tasks

Across the ABCD, AHRB, and MLS studies, reward processing was measured usingcomparable versions of the MID task. The MID task (Knutson et al., 2000) is used to model BOLDsignatures of the anticipation and receipt of monetary gains or losses. TheMID task and their nuanced differences across the ABCD, AHRB, and MLSstudies are described inSupplemental Section 1.2. The focus of the present work is onthe anticipatory phase of the task.

#### 2.2.2 MRI acquisition details

The acquisition details for the ABCD, AHRB, and MLS datasets are summarizedin Supplemental Section 1.3Table S2.

#### 2.2.3 Data quality control and preprocessing

First, quantitative metrics reported from MRIQC version 23.1.0 (Esteban et al., 2023) for thestructural and BOLD data are evaluated to assess data quality andpotentially problematic subjects. Second, behavioral data were inspected toconfirm that participants have the behavioral data for each run and thatparticipants performed at the targeted probe hit rate (e.g., at or near 60%overall probe hit rate, seeSupplemental Section 1.2). Then, structural andfunctional MRI preprocessing is performed using fMRIPrep v23.1.4 (Esteban, Markiewicz, Goncalves, et al.,2022; RRID:SCR_016216), which is based on Nipype 1.8.3 (Esteban, Markiewicz, Burns, et al.,2022; RRID:SCR_002502) and the results are inspected to confirmno subjects’ preprocessing steps failed.

Preprocessing between ABCD, AHRB, and MLS is held constant except for twodifferences. First, the MLS datasets did not collect fieldmaps and therepetition time for MLS (2,000 ms) is slower than the repetition time (800ms) in ABCD/AHRB. Therefore, fMRIPrep’s fieldmap-less distortioncorrection (SyN-SDC) is used to estimate and correct for fieldmapdistortions in MLS and slice-timing correction is applied*only*on the MLS data. For the ABCD and AHRB data,fieldmap-less distortion correction is used*only*when asubject does not have the necessary fieldmaps. Outside of these twoexceptions, the preprocessing of the BIDS data was preprocessed usingidentical pipelines. The complete preprocessing details are included inSupplemental Section1.4.

### 2.3 Analyses

This project is focused on the effects of analytic decisions on estimates ofreliability across (run/session) measurement occasions in task fMRI. As areminder, reliability is the estimate of how similar two measures (in this case,voxels for a given contrast from an fMRI 3D volume) are in terms of estimatedeffects (continuous) and/or the presence/absence of a significant effect(binary). We distinguish individual and group estimates inFigure 1and describe the calculations below. For thecontinuous estimates of reliability described below, the analyses will beperformed separately on task voxels that exceed and do not exceed an*apriori*specified threshold applied on the NeuroVault (Gorgolewski et al., 2015) meta-analysiscollection that comprises the anticipatory win phase across 15 whole brain mapsfor the MID task (Wilson et al., 2018;Collection: 4258, Image ID: 68843). The*suprathreshold*task-positive voxels are those that exceed the threshold (*z*> 3.1) and the*subthreshold*task voxels are those thatdo not exceed the threshold (*z*< 3.1) in the map. Weacknowledge that the threshold of*z = *3.1 is arbitrary(uncorrected,*p*-value = .001) and that the voxels thatfall below and above this threshold may not be significantly different (Gelman & Stern, 2006). However, toconstrain the problem space, this is a researcher’s decision that is madein these analyses (Gelman & Loken,2014;Simmons et al.,2011).

**Fig. 1.**
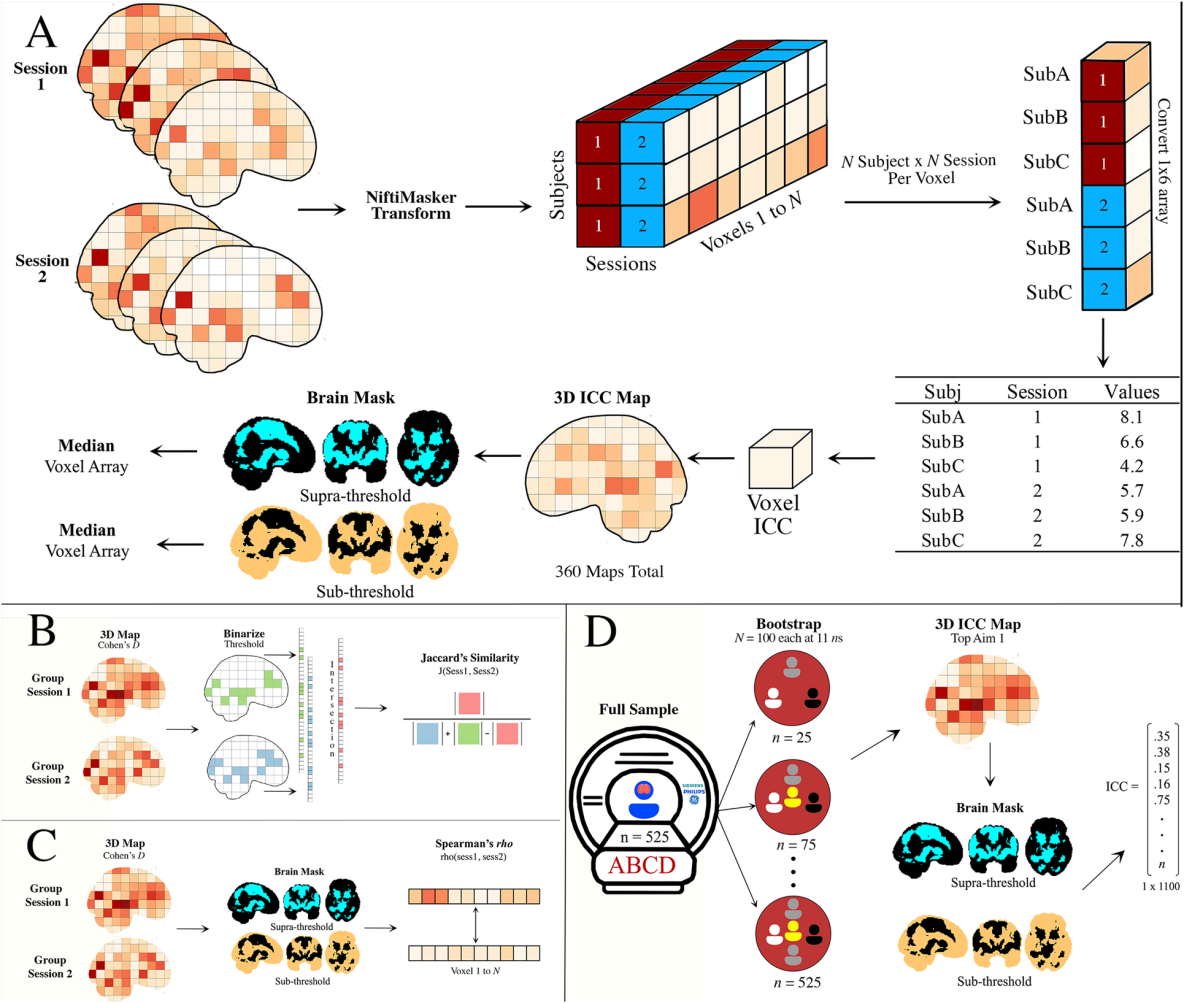
Diagram of (A) continuous (individual), (B/C) binary/continuous (group),and (D) random subsampling of estimates of reliability acrossmeasurement occasions in 3D volumes of fMRI data. Group = groupaverage of activation; Sub = subject; ICC = intraclasscorrelation; supra- and subthreshold mask is >3.1 of NeuroVaultVault Image ID #68843 (Collection #4258).

#### 2.3.1 Descriptive statistics

The mean, standard deviation, count, and frequencies are reported fordemographic variables from the ABCD, AHRB, and MLS datasets. For ABCD, AHRB,and MLS, participants self-reported on age, sex, and race/ethnicity. ABCD:sex is reported as sex at birth (male, female, other, or not reported);race/ethnicity is reported on a five-item scale: White, Black, Hispanic,Asian, Other. AHRB: sex is reported as sex at birth (male or female);race/ethnicity is available on a four-item scale: White, Non-Hispanic,Black, Non-Hispanic, Hispanic/Latinx, Other. MLS: sex is reported as sex atbirth; race is available on an eight-item scale: Caucasian, AfricanAmerican, Native American, Asian American, Filipino or Pacific Islander,Bi-Racial, Hispanic Caucasian, and Other.

Behavioral data from the MID task, such as the mean and distribution of probehit rate and mean response times (RT) across subjects, will be reported asSupplemental Information. The task design is programmed to achieve a probehit rate of approximately 60% for each subject. It should be noted that theRT for the probe is not consistently collected across the ABCD, AHRB, andMLS datasets.

#### 2.3.2 Impact of analytic decisions on reliability in fMRI data

First-, second-, and group-level analyses are performed using Python 3.9.7and Nilearn 0.9.2 (Abraham et al.,2014). Details about these three analytic steps are describedbelow and the code is provided on Github (Demidenko, Mumford, & Poldrack, 2024b). As listed inTable 1and the described next,the analytic decisions will be limited to the first-level analysis.

*Analytic Decisions*: For reasons described in theintroduction, the focus of analytic decisions in this paper will be on**four**categories: Smoothing, Motion Correction, TaskContrast, and Task Parametrization. As reported in empirical studies andmeta-analyses of task fMRI reliability (Bennett & Miller, 2010;Caceres et al., 2009), one way to improve reliability of fMRIdata is by increasing the signal-to-noise ratio in the BOLD data throughdifferent smoothing kernels (Caceres et al.,2009), reducing motion effects in the fMRI data (Gorgolewski et al., 2013;Kennedy et al., 2022) and using taskdesigns/contrasts that evoke increased neural activity (Han et al., 2022;Kennedy et al., 2022). These analytic decisions are described ingreater detail inSupplemental Section 1.1.

*Within-run Analysis:*A general linear model (GLM) is fitusing Nilearn (e.g.,*FirstLevelModel*) to estimate theresponse to task-relevant conditions in the BOLD timeseries for eachparticipant/voxel. The BOLD timeseries are masked and spatially smoothedusing specified full-width half-maximum (FWHM) Gaussian kernel options (see“Smoothing” inTable1) and the timeseries are prewhitened using an “ar1”noise model. A GLM is fit (using*FirstLevelModel*) for adesign matrix that includes the 15 task-relevant regressors (see taskdetails inSupplementalSection 1.2) and a set of nuisance regressors. Depending on thedecision criteria (see “Motion Correction” inTable 1), nuisance regressors mayinclude, for example, (**A**) estimated translation and rotation(+ derivatives) of head motion or**A**+ first eightaCompCor noise components and the corresponding cosine regressors for highpass filtering (with a cutoff of 128 seconds) that are calculated byfMRIPrep (see preprocessing of functional data). Task regressors areconvolved with the SPM hemodynamic response function (HRF). The resultingbeta estimates from the GLM, for each individual subject and run, are usedto compute four contrasts for the MID task (see “TaskContrasts” inTable 1).

*Within-session Analysis*: Per subject, each study collectedtwo runs for each of two sessions. For each of the four contrast types, thebeta and variances estimates from the two MID runs for each subject areaveraged using Nilearn’s precision-weighted fixed effects model(i.e.,*compute_fixed_effects*).

*Group-level Analysis (within-session)*: The MID task-weightedfixed effects contrast files are used in a group-level mixed effect model(i.e., Nilearn’s*SecondLevelModel*) to average thewithin-subject estimates across subjects. These group maps are used asmeasures of the average activation patterns during the MID task in each ofthe studies across each of the four contrast types within each session.

The resulting individual and group maps from the four contrasts are used incalculating two different estimates of reliability (described in detailbelow). First, the resulting*within-run analysis*maps(i.e., for each run) are used for the continuous estimate of reliability*within*each session (i.e., reliability across runs).Then, the resulting*within-session analysis*maps, computedfrom the weighted fixed effects model, are used in the continuous estimateof reliability*between*the two sessions. Due to thetemporal difference within and between sessions, the reliability withinsessions would be hypothesized to be greater than that of between sessions.The resulting group-level analysis maps are used in the binary estimate ofreliability*between*sessions.

#### 2.3.3 Individual estimates of reliability: Intraclass correlation for continuous outcomes

Reliability for continuous outcomes at the individual level is estimatedusing ICC. The ICC is an estimate of between-subject and within-subjectvariance that summarizes how similar the signal intensities are for a givenvoxel from a 3D volume across sessions. As described inLiljequist et al. (2019), there are severalversions of the ICC, which vary in whether the subjects and sessions areconsidered to be fixed (e.g., ICC[1]), subjects are considered to be randomand sessions are considered to be fixed (e.g., consistency, estimated viaICC[3,1]), or the subjects and sessions are considered to be random (e.g.,agreement, estimated via ICC[2,1]). In the case of these analyses, we assumethat subjects are random but do not assume that sessions are random for tworeasons. First, in the case of reliability of runs within a session, theruns are administered in a fixed manner and the state of the participantcannot be assumed to be random for each. Second, in the case of reliabilityacross sessions, during the follow-up session subjects have experienced theMRI environment and the task design in the scanner. In this case, again, itis difficult to assume that sessions are in fact random as the practice andsession effects may be present. Thus, we estimate the consistency (ICC[3,1])of the signal intensity for a given voxel across measurement occasions.

Several packages exist to calculate ICC and Jaccard/Dice coefficients. Forexample,*ICC_rep_anova*&*Similarity*in Python (Gorgolewski et al., 2011),*fmreli*in MATLAB (Fröhner et al., 2019), and*3dICC*in AFNI(Chen et al., 2017). However,these packages are either (a) limited to a specific ICC calculation (e.g.,ICC[3,1]), (b) not easy to integrate into reproducible python code (e.g.,*fmreli*), (c) do not include similarity calculations(e.g.,*3dICC*), or do not return information aboutbetween-subject, within-subject, and between-measure variance components.Thus, to have the flexibility to estimate ICC(1), ICC(2,1), and ICC(3,1),Dice and Jaccard similarity coefficients, and Spearman correlationssimultaneously, we wrote and released an open-source Python package withreliability and similarity functions that works on 3D NifTi fMRI images.

The*PyReliMRI*v2.1.0 (Demidenko, Mumford, & Poldrack, 2024a) Python package isused to calculate continuous estimates of reliability.*PyReliMRI*implements a voxel-wise ICC calculation(e.g.,*voxelwise_icc*) for 3D NIfTI images between runsand/or between sessions (see the ICC example in study flowchart;Fig. 1A). The function takes in a list oflists (e.g., list of session 1 and list of session 2) of ordered paths tothe preprocessed data [in MNI space] for session 1 (or run 1) and session 2(or run 2) subjects, and a binary [MNI space] brain mask. The package isflexible to take in more than two sessions (or runs). An ICC type option(e.g., “icc_1,” “icc_2,” or“icc_3”) indicates the type of ICC estimate that is calculatedacross the voxels within the masked 3D volume. The function returns adictionary with five separate 3D volumes containing the voxel-wise (1) ICCestimate, (2) lower bound ICC, (3) upper bound ICC, (4) between-subjectvariance (BS), and (5) within-subject variance (WS) and, in case ofICC(2,1), (5) between-measure variance, or the measurement additive bias.Like the ICC and 95% confidence calculation in the*pingouin*package (Vallat, 2018), the ICCconfidence interval in*PyReliMRI*is calculated using the*f-*statistic (Bonett,2002) to reduce the computation time compared with usingbootstrapped estimates.

$$ICC(3,1)\;=\;\frac{MSBS\;-\;MSError}{MSBS\;+\;MSError}=\frac{\sigma_{r}^{2}}{\sigma_{r}^{2}+\sigma_{v}^{2}}$$ (Eq. 1)

*Aim 1a*evaluated the effect of analytic decisions (seeTable 1;Fig. 1A) on the ICC(3,1) (Equation 1for two measurement occasions) for individual[continuous] estimates of voxel activity across the ABCD, AHRB, and MLSstudies. The parameters inEquation1are:*MSBS*is the mean squared between-subjecterror and*MSError*is the mean squared error. As describedinLiljequist et al. (2019), thedifferences in the numerator is the between-subject variance($\sigma_{r}^{2}$)and the denominator is the sum of the between-subject variance($\sigma_{r}^{2}$)and the within-subject variance (or noise,[$\sigma_{v}^{2}$]).For each study,*voxelwise_icc*within the*brain_icc.py*script is used to estimate the voxel-wiseICC(3,1) for between-run and between-session reliability across the 360model permutations. First, voxel-wise average and standard deviation fromthe resulting ICCs for the 360 model permutations are reported in two 3Dvolumes. Second, the range and distribution of median ICCs across each study(three) and analytic decision category (four) are plotted acrosssuprathreshold task-positive and subthreshold ICCs using Rainclouds (Allen et al., 2019) and the median andstandard deviation are reported in a table. Third, to visualize the orderedmedian ICCs across the 360 model permutations for suprathresholdtask-positive and subthreshold ICCs, specification curve analyses are used(Simonsohn et al., 2020).Specifically, results across the 360 model permutations are reported using aspecification curve to represent the range of estimated effects across thevariable permutations. This consists of two panels: Panel A represents the*ordered*median ICC coefficients and the associated 95%confidence interval (across samples) colored based on no significance(gray), negative (red), or positive (blue) significance from the Null (Nullhere is 0) and Panel B represents the analytic decisions from each of thefour categories (seeTable 1) thatproduced the median ICC estimates. The median ICC estimates from the 360models are reported separately for suprathreshold task-positive andsubthreshold activation (the specification curve for all ICC estimates forsuprathreshold task-positive and subthreshold activation is provided asSupplementalInformation). Finally, to evaluate the effect of the analyticdecisions on the median ICC, hierarchical linear modeling (HLM) is performedas implemented in the*lmer()*function from the*lme4*R package (Bates etal., 2020). HLM is used to regress the median ICC on the [four]analytic decisions as fixed effects with a random intercept model are fit(Matuschek et al., 2017) forsamples across the suprathreshold task-positive and subthreshold maps.Multiple comparisons corrections are applied using the Tukey adjustment asimplemented in the*emmeans*package (Lenth et al., 2023). For these HLM models, theinterpretation focuses on the significant, nonzero effect of an independentvariable (e.g., smoothing) on the dependent variable (e.g., median ICC),while the remaining independent variables are assumed to be zero.

*Aim 2*evaluated the change in between- and within-subjectvariance across the analytic model permutations. Similar to Aim 1 (Fig. 1A),*voxelwise_icc*within the*brain_icc.py*script is used to estimate the BSand WS across the 360 model permutations. The range and distribution ofmedian BS and WS across each study and analytic decision category areplotted across suprathreshold task-positive and subthreshold BS/WS usingRainclouds. Then, two separate specification curve analyses report the*ordered*median BS and WS coefficients in one panel andthe analytic decisions that produced the BS and WS estimates in a secondpanel separately for suprathreshold task-positive and subthresholdactivation. Finally, like Aim 1, two HLMs are used to regress the median BSand median WS on the [four] analytic decisions as fixed effects with arandom intercept only for sample across the suprathreshold task-positive andsubthreshold maps. Multiple comparisons corrections are applied using theTukey adjustment. Like Aim 1, the interpretation focuses on the significant,nonzero effect of an independent variable (e.g., smoothing) on the dependentvariable (e.g., median BS or median WS), while the remaining independentvariables are assumed to be zero.

*Aim 3*evaluated the sample size at which the ICC stabilizes(Fig. 1D). The chosen pipeline isbased on the highest median ICC across the studies for the suprathresholdtask-positive mask from Aim 1a and is rerun for the ABCD sample. Based onthis pipeline, the first-level analysis steps are repeated for N =525 from the N = 2,000 subsample for only the ABCD data. Then,*voxelwise_icc*within the*brain_icc.py*script is used to derive estimates of the median ICC, BS, and WS for thebetween-runs (e.g., measurement occasions) reliability across randomlysampled subjects for 25 to 525 subjects in intervals of 50. Similar to themethods inLiu et al. (2023), 100iterations are performed at each N (with replacement) and the median ICC,the associated BS and WS estimates are retained from*voxelwise_icc*. The average and 95% confidence intervalfor the estimates across the 100 iterations are plotted for each interval of*N*with the y-axis representing the median ICC andx-axis representing*N*. The plotted values will be used toinfer change and stability in the estimated median ICCs and variancecomponents across the sample size. If stability is not achieved by*N*= 500, the sample is extended to*N*= 1,000 and the analyses are repeated.

#### 2.3.4 Group estimates of reliability: Jaccard coefficient for binary and spearman correlation for continuous outcomes

The estimate of reliability for group analyses is evaluated using the Jaccardsimilarity for binary and Spearman correlation for continuous outcomes. Theestimates are used to evaluate how the MID task evokes BOLD activation abovea prespecified threshold (*p*< .001) in the samevoxels for*groups*of subjects across measurement occasions(run/session) in the ABCD, AHRB, and MLS studies.

The*PyReliMRI*package is used.*PyReliMRI*calculates the similarity between two 3D volumes using a Jaccard’scoefficient which, in short, is the intersection divided by the unionbetween two binary images (seeFig. 1B)or the Spearman correlation, which is ranked correlation between twocontinuous variables (seeFig. 1C). TheJaccard coefficient ranges from 0 to 1, whereby higher values reflectgreater similarity between two images. Like the product–momentcorrelation, the Spearman correlation ranges from -1 to 1, whereby values>0 indicate a positive association between images and values<0 indicate a negative association between images. The function(i.e.,*image_similarity*) takes in the paths for MNI*image file1*and*image file2*, aspecified MNI mask and integer (i.e., z-stat/t-stat) at which to thresholdthe image. The images are masked (if a mask is provided), thresholded at thespecified integer (if a threshold is provided), and the resulting images arebinarized per user’s input (i.e., if threshold = 0, theresulting similarity = 1). Based on the specified similarity metric,the resulting estimates are similarity (e.g., Dice/Jaccard) or correlationcoefficient (e.g., Spearman) between the two 3D NIfTI images. For similaritybetween 2 + NIfTI images,*pairwise_similarity*isused. Similar to*image_similarity*,*pairwise_similaity*takes in paths for an MNI mask, athreshold integer for the 3D volumes and the similarity type. Unlike*image_similarity*,*pairwise_similarity*allows for a list (2+) of paths pointing to 3D volumes and createspair-wise combinations across the image paths between which to estimatesimilarity. The function returns the similarity coefficient in a data framewith the resulting similarity (or correlation coefficient) and the imagelabel (e.g., base name of the provided path for given volume).

$$J(A,B)\;=\;\frac{\left|A\;\cap \;B\right|}{\left|A\;\cup \;B\right|}$$ (Eq. 2)

$$Spearman\;Correlation_{A,B}\;=\;\frac{6\Sigma d_{i}^{2}}{n(n^{2}\;\;-1\;)}$$ (Eq. 3)

*Aim 1b*evaluated the effect of analytic decisions (seeTable 1) in the Jaccard’ssimilarity coefficient (Equation 2;Fig. 1B) and Spearman correlation(Equation 3;Fig. 1C) using the group binary andcontinuous estimates. InEquation 2,J(A, B) is the similarity coefficient between A (session 1) and B (session2). This is derived from intersection, |A ∩ B|, which represents theelements that are common to both A and B divided by the union, |A ∪B|, or the elements that are both in A and/or B. InEquation 3, the Spearman rank coefficient, asimplemented in Scipy stats using*spearmanr*(Virtanen et al., 2020), is ranked correlationbetween unthresholded images A and B, whereby Σd² is the sumof squared differences between ranked values in sessions A and B, normalizedby (n * (n² – 1)).

Since the Jaccard similarity coefficient is sensitive to thresholding andsample size (Bennett & Miller,2010), in Aim 1b an equal sample size (e.g., N ~ 60^‡^) is chosen for eachstudy to compare how the similarity between sessions varies across studies.For all 360 pipelines, a group-level (average) activation map is estimatedfor each session. In the case of the Jaccard coefficient, the group maps arethresholded at*p*< .001. In the case of the Spearmancoefficient, the group maps are masked using a suprathreshold task-positivemap from NeuroVault (https://identifiers.org/neurovault.collection:4258; Image ID:68843). Then, the paths for the pipelines and sessions are called using the*pairwise_similarity*within the*similarity.py*script. The resulting coefficients reportthe similarity between analytic pipelines and sessions for each study. Foreach study, the coefficients are plotted to reflect the distribution andrange of coefficients. Both Jaccard’s and Spearman correlation arereported separately. Like Aim 1a and Aim 2, two HLMs are used to regress theJaccard coefficients and Spearman correlation on the [four] analyticdecisions nested within study. Multiple comparisons corrections are appliedusing the Tukey adjustment.

## 3 Results

Given the breadth of the analyses (seeTable1), the results in the main text focus on Session 1 between-runindividual- and group-level reliability estimates for the suprathreshold mask.Differences are briefly noted for between-session reliability estimates andsubthreshold models and are reported in detail in theSupplemental Materials.

As permitted, aggregate and individual subjects’ data are made publiclyavailable on NeuroVault (Gorgolewski et al.,2015) and/or OpenNeuro (Markiewicz etal., 2021). The complete set of group-level and ICC maps are publiclyavailable on Neurovault for ABCD (6,180 images;https://identifiers.org/neurovault.collection:17171), AHRB (2,400 images;https://identifiers.org/neurovault.collection:16605), and MLS (2,400images;https://identifiers.org/neurovault.collection:16606). For each run andsession, the BIDS input data and derivations for MRIQC v23.1.0 and fMRIPrep v23.1.4are available on OpenNeuro for AHRB (Demidenko,Huntley, et al., 2024) and MLS (Demidenko, Klaus, et al., 2024). Since the ABCD data are governed by astrict data use agreement (March 2024), the processed data will be made publiclyavailable via the NDA (DOI:10.15154/3wqz-gd77) at a later date as part of the ABCC release. Thefinal code for all analyses is publicly available on Github (Demidenko, Mumford, & Poldrack, 2024b).

In the Supplemental Information of the Stage 1 submission, we stated that we wouldadjust the smoothing weight for the MLS as its voxel size, 4 mm anisotropic, wouldresult in greater inherent smoothness of the data than ABCD/AHRB samples (2.4 mmisotropic voxel). A weight of .50 was applied to the smoothing kernels of the MLSdata. This resulted in 3.6, 4.8, 6.0, 7.2, and 8.4 mm smoothing kernels for theAHRB/ABCD data and 3.0, 4.0, 5.0, 6.0, and 7.0 mm smoothing kernels for the MLS data(Fig. S4). In theresults, the MLS ordinal values are relabeled to map onto the values used forAHRB/ABCD for reporting purposes.

### 3.1 Deviations from Stage 1 Registered Report

There are one moderate and two minor deviations from the Stage 1 RegisteredReport (https://doi.org/10.17605/OSF.IO/NQGEH). First, fieldmap-lessdistortion correction is not applied on the MLS data because the data werecollected using spiral acquisition. The ABCC data select a single fieldmapwithin a session to apply on*all*of the functional runs, sosubjects without a fieldmap folder are excluded and fieldmap-less distortioncorrection is not used on the ABCD data. In AHRB, fieldmap-less distortioncorrection was used for only*one*subject. Second, in Aim 1b weproposed to use thresholded images (e.g.,*p*< .001,approx.*t*> 3.2) to estimate the Jaccard/Spearmansimilarity between the model permutations for the estimated group maps. However,this statistic is arbitrarily sensitive to differences in the number of modelpermutations when subjects are excluded in cases of failed preprocessingfeatures, such as aCompCor mask errors. To improve the interpretability of thesimilarity estimates across analyses with different number of includedobservations (seeSupplemental Fig. S3), we converted all*t*-statisticgroup maps to Cohen’s*d*effect size maps using theformula:$\frac{t-statistic}{\sqrt{N}}$.Cohen’s*d*= .40 is used as the alternativethreshold for Aim 1b as for preregistered N ~ 60 a conversion of*t-statistic*= 3.2 would be near this threshold.Third, the analyses proposed to evaluate 360 analytic decisions across the threesamples. However, no subjects in the final AHRB and MLS samples exceeded mean FD= .9 so it was not possible to perform Motion option 5 (Motion option 3+ exclude mean FD ≥ .9) or Motion option 6 (Motion option 4+ exclude mean FD ≥ .9). As a result, the model permutations arerestricted to 240 permutations (5 = FWHM, 6 → 4 = Motion; 3= Model Parameterization; 4 = Contrasts) with relevant data acrossthe 3 samples and are the focus of the below analyses.

### 3.2 Descriptive statistics

For Aim 1 and Aim 2, the final sample sizes from the University of Michigan siteare 119 for the ABCD sample, 60 for the AHRB sample, and 81 for the MLS sample.These participants met the criteria of having a mean FD < .90, completedat least two sessions with two runs each, had available behavioral data, andpassed quality control. For*N*= 15 subjects in the ABCDsample, aCompCor ROIs failed, but otherwise the data passed QC and so thesesubjects were not excluded in Motion option3 and option4 models that include thetop-8 aCompCor components as regressors. The final random subsample from theBaseline ABCD data for Aim 3 is*N*= 525.

Demographic information across the 3 samples for Aim 1 and Aim 2 (ABCD =119; AHRB = 60; MLS = 81) is reported inSupplemental Table S4.The average number of days between sessions is largest for the MLS sample (1,090days), followed by ABCD (747 days) and AHRB (419 days;Fig. S5). On average,mean FD was higher in the ABCD sample versus the AHRB and MLS samples (Fig. S6;Table S5). The samplesalso differed on average response probe accuracy (%), whereby on average MLSparticipants had a higher and faster probe response accuracy than ABCD and AHRBsamples.

The estimated model efficiency, defined as$Efficiency\;=\;\frac{1}{c{\left(X\prime X\right)}^{-1}c\prime }$,varied as a function of Model Parameterization and Contrast types across thethree samples (seeFig.S7). The Anticipation Model (i.e., onset times locked to Cue onsetand duration the combined duration of Cue and Fixation cross) was consistentlyestimated to be the most efficient model across the three samples for the*Large Gain*versus*Neutral*and*Small Gain*versus*Neutral*contrasts.

### 3.3 Aim 1a: Effect of analytic decisions on median ICC estimates for individual continuous maps

**Fig. 2.**
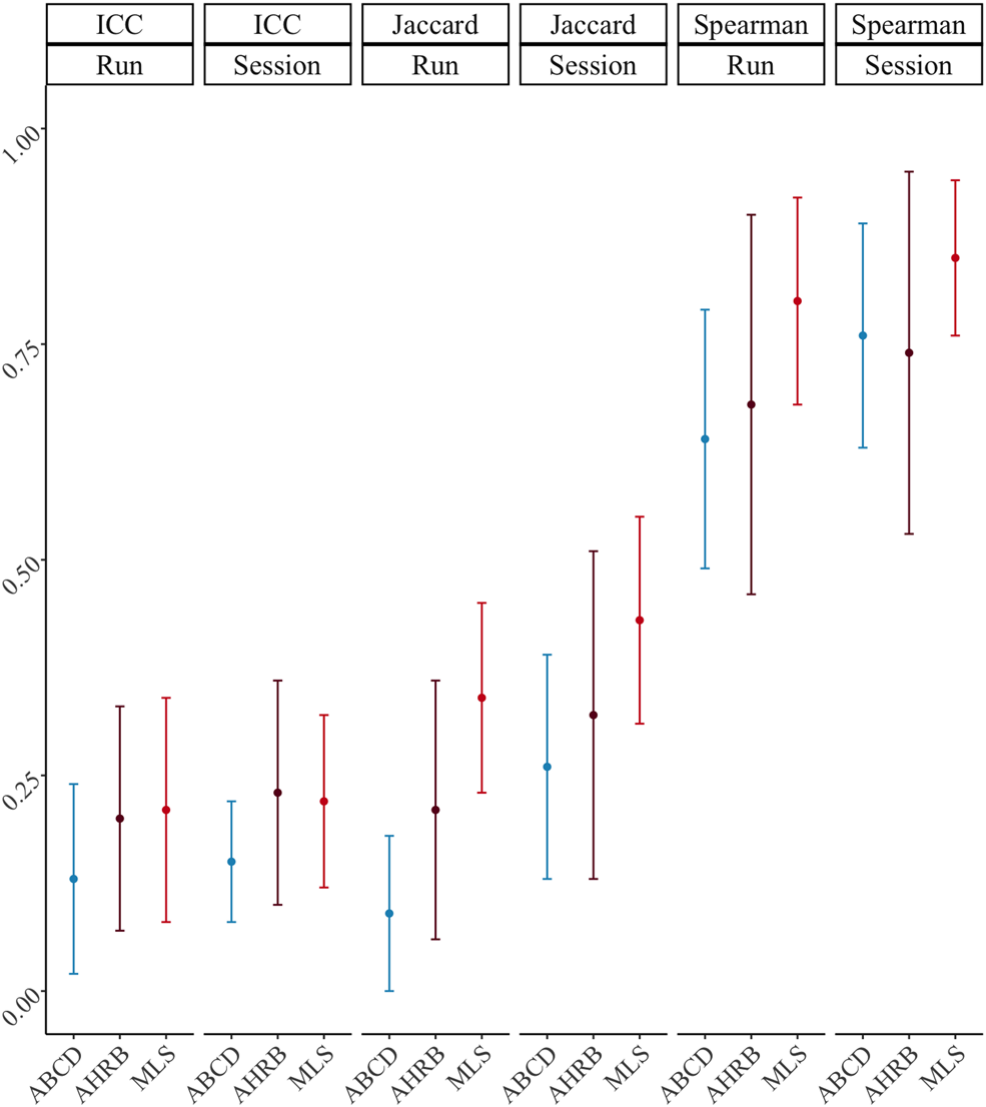
Session 1 between runs and between sessions: Mean +/- 1 standarddeviation (SD) of suprathreshold median intraclass correlationcoefficient (ICC), Jaccard and Spearman similarity coefficients from 240analytic models across ABCD, AHRB, and MLS samples.*Note:*Estimates inSupplemental TableS5.

Aim 1a proposed to evaluate the estimated individual map similarity betweenmeasurement occasions (runs/sessions) using the ICC(3,1) across 240 pipelinepermutations. InTableS5(Fig. 2), the medianbetween-run Session 1 ICCs are slightly lower than the between-session ICCs(between-run: ABCD = .11 [range: -.04 –.43]; AHRB = .18 [range: .00 – .52]; MLS = .18 [range: .04– .55];between-session: ABCD = .15 [range:.03 – .34]; AHRB = .21 [range: .04 – .53]; MLS = .21[range: .06 – .47]). The mean and standard deviation of the 3D volumesacross the 240 analytic decisions are reported inSupplemental Figure S8.Across the three samples, a consistent pattern is observed, whereby the regionswith the highest ICCs, on average, are within the visual and motor regions.Notably, the lowest ICCs, on average, are within the ventricles and whitematter. The suprathreshold distribution of the median estimates across the fourmodel options and three samples is reported inFigure 3and the specification curve of the median ICC estimates isreported inFigure 4. Note, thesubthreshold reported inSupplemental Figure S10.

**Fig. 3.**
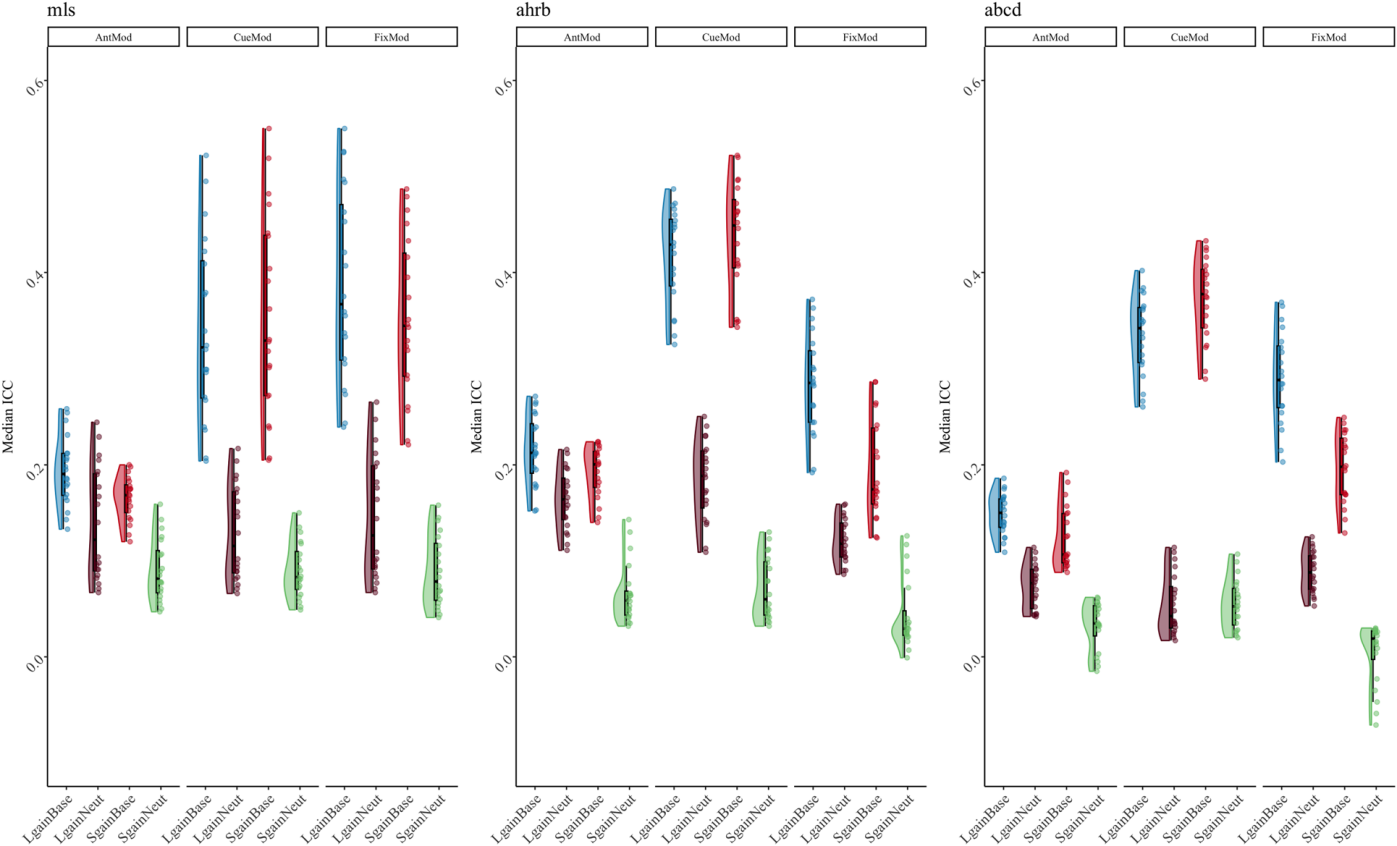
Suprathreshold median ICC Session 1 between-run reliability estimates forContrast (con) and Model Parameterization analytic options across theABCD, AHRB, and MLS samples. Complete distribution across four analyticoptions inSupplemental Figure S9.

**Fig. 4.**
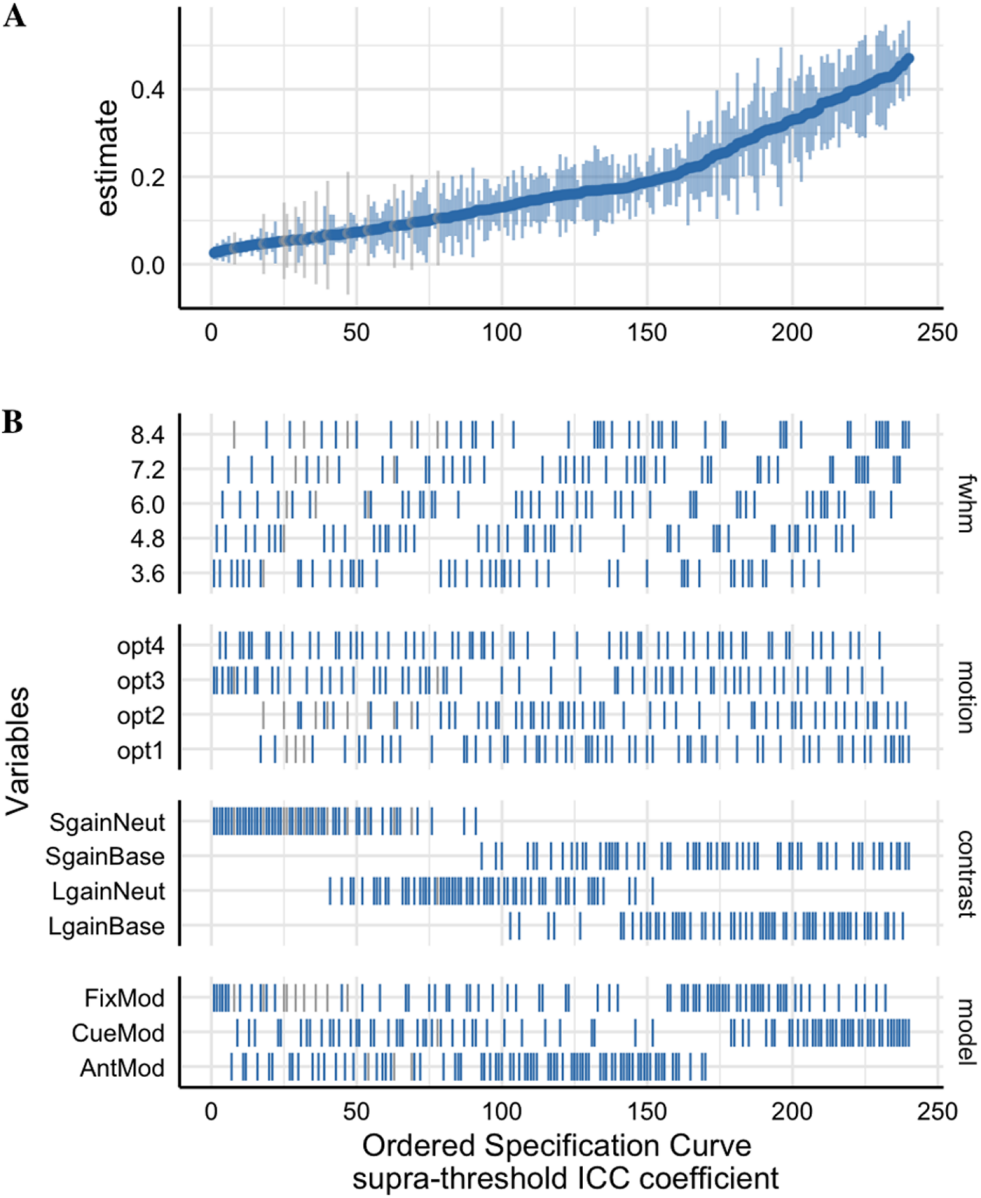
The suprathreshold specification curve of the Session 1 between-runmedian ICC estimates across 240 pipeline permutations for the ABCD,AHRB, and MLS samples. Full length of estimates reported inFigure S11. (A)The distribution of the point estimate (average) and distribution (errorbars) across the three samples. (B) The model options (four) associatedwith each estimate.

The effects reported inFigures 3and4illustrate that the largest differences inthe median ICC estimate are associated with model parameterization and thecontrast type. Even though the Anticipation Model (“AntModel”) hasthe highest estimated contrast efficiency within each sample, contrary to ourhypothesis, the highest median ICC is associated with the Cue Model(“CueMod”) in which the onset and duration are locked to the cuestimulus. However, using an interaction to probe the distributions inFigure 3, post hoc analyses suggest that theCue Model finding is largely driven by the Implicit Baseline contrasts (see Aim1b).Supplemental FigureS12, of the Model Parameterization-by-Contrast, suggests negligibledifferences between Model Parameterization for the contrast of the Neutralcontrasts.

Independent of model parameterization and consistent with our hypothesis andprevious reports in the task fMRI literature (Han et al., 2022;Kennedy et al.,2022), the highest median ICC is consistently observed for the*Large Gain*versus*Implicit Baseline*contrast. In line with the reported estimates shown inFigures 3and4, theHLM model for the suprathreshold mask shows a significant association betweendifferent FWHM, Motion, Model Parameterization, and Contrasts model optionscompared with their respective reference values (Table 2). Specifically, the median ICC estimates increasedwith larger smoothing kernels and decreased with more stringent motioncorrection. Additionally, primarily driven by the*ImplicitBaseline*conditions, median ICC for “CueMod” and“FixMod” increased in comparison with the “AntMod”(see interaction plot inFig. S12). Last, median ICC decreased in comparison with the*Large Gain*versus*Implicit Baseline*contrast. For example, the contrast*Large Gain*versus*Neutral*has a median ICC that is .17 lower, on average,compared with the*Implicit Baseline*contrast when holding otherdecisions constant (see marginal means comparisons inSupplemental Table S6).While most parameters are significant inTable2, the effects vary in their relative importance in the model. Thevariability in the median ICC estimate across 240 pipelines and 3 samples isbest explained by contrast (marginal ∆R^2^: .55) and modelparameterization (marginal ∆R^2^: .10). FWHM and motion had asmaller impact on ∆R^2^, .03 and .03, respectively. In fact,including aCompCor components (Motion option 3) and aCompCor components +censoring high motion volumes (Motion option 4) is associated with a slightdecrease in the median ICC estimate as compared with no motion correction(Motion option 1), b = -.05 and b = -.05, respectively. A similarfinding is observed for the subthreshold mask, whereby the contrast(∆R^2^: .56) and model parameterization(∆R^2^: .10) decision had a larger impact on∆R^2^than the FWHM (∆R^2^: .04) or motion(∆R^2^: .02) decisions (seeFig. S14;Table S7). In general,the voxel-wise distribution of ICC estimates tends to be higher for thesuprathreshold mask than for the subthreshold masks (seeSupplemental Fig. S14).Interpretations are generally consistent for between-session median ICCestimates across the 240 pipeline permutations (seeTable S9andFigs. S18, S19).

**Table 2.** Hierarchical linear model: (A) Linear associations between the analyticdecisions and the*Session 1 between-run median IntraclassCorrelation Coefficient (ICC[3,1]), Between-subject (BS) andWithin-subject variance (WS) from suprathreshold mask*and(B) the impact of the analytic category on the marginalR^2^.

**A. HLM Estimates for Suprathreshold Mask**												
	Median ICC(3,1)				Median BS				Median WS			
*Predictors*	*b*	*CI*		*p*	*b*	*CI*		*p*	*b*	*CI*		*p*
(Intercept)	.23	.20 – .26		<.001	.27	.18 – .35		<.001	.91	.72 – 1.10		<.001
Reference [3.6]												
fwhm [4.8]	.02	.01 – .04		.003	-.03	-.06 – .00		.09	-.23	-.28 – -.18		<.001
fwhm [6.0]	.04	.03 – .06		<.001	-.04	-.07 – -.01		.003	-.36	-.41 – -.31		<.001
fwhm [7.2]	.06	.04 – .07		<.001	-.06	-.09 – -.03		<.001	-.44	-.49 – -.39		<.001
fwhm [8.4]	.07	.05 – .08		<.001	-.07	-.10 – -.04		<.001	-.49	-.54 – -.44		<.001
Reference [opt1]												
motion [opt2]	-.01	-.03 – .00		.07	-.04	-.06 – -.01		.01	-.14	-.18 – -.09		<.001
motion [opt3]	-.05	-.06 – -.04		<.001	-.10	-.13 – -.08		<.001	-.23	-.28 – -.19		<.001
motion [opt4]	-.05	-.06 – -.03		<.001	-.10	-.13 – -.08		<.001	-.24	-.28 – -.20		<.001
Reference [AntMod]												
model [CueMod]	.10	.09 – .11		<.001	.15	.13 – .17		<.001	.26	.23 – .30		<.001
model [FixMod]	.05	.04 – .06		<.001	.12	.10 – .14		<.001	.27	.23 – .31		<.001
Reference [LgainBase]												
con [LgainNeut]	-.17	-.18 – -.16		<.001	-.22	-.25 – -.19		<.001	-.28	-.32 – -.23		<.001
con [SgainBase]	-.02	-.04 – -.01		<.001	-.02	-.05 – .00		.09	.00	-.04 – .05		.93
con [SgainNeut]	-.23	-.24 – -.22		<.001	-.24	-.27 – -.21		<.001	-.31	-.35 – -.26		<.001

**B. Analytic Category Model Impact**												
Comparison	χ ^2^	Orig R ^2^	New R ^2^	∆R ^2^	χ ^2^	Orig R ^2^	New R ^2^	∆R ^2^	χ ^2^	Orig R ^2^	New R ^2^	∆R ^2^
[Full] vs. [New – fwhm]	95	.72	.69	.03	25	.47	.45	.02	384	.52	.31	.21
[Full] vs. [New – motion]	81	.72	.69	.03	81	.47	.42	.05	138	.52	.46	.06
[Full] vs. [New – model]	263	.72	.62	.10	162	.47	.37	.10	221	.52	.42	.10
[Full] vs. [New – con]	864	.72	.17	.55	397	.47	.17	.30	285	.52	.38	.14

We had hypothesized that the ICC estimates in the older samples (AHRB/MLS) wouldmeaningfully differ from those in the younger sample (ABCD). Overall, ICCestimates were higher in the older than in the younger sample for*between-run*,*t*(497.2) = 5.53,*p*< .001,*d = *.43, and*between-session*,*t*(669.9) = 9.57,*p*< .001,*d = *.66.

#### 3.3.1 Summary of findings for Aim 1a

Overall, between-run ICCs are slightly lower than between-session ICCs.Across the three samples, the highest ICCs, on average, are within visualand motor areas and the lowest ICCs are within the ventricles and whitematter. InTable 1, it washypothesized that the optimal analytic decisions would be FWHM Smoothing2.5x the voxel size, Motion correction that includes translation/rotation,their derivatives, the first 8 aCompCor components and exclusion of>.90 mFD subjects, the anticipation Model Parameterization, andContrast*Large Gain*>*ImplicitBaseline*. Contrary to registered hypotheses: (1) smoothing hada small but linear effect on ICC estimates, whereby the largest median ICCwas for the largest FWHM smoothing kernel (3.5x voxel size); (2) Motioncorrection had minimal and negative impact on median ICCs in case of morerigorous corrections; and (3) the Cue and Fixation Models had higherestimated median ICCs than the Anticipation model.*Post hoc*analyses illustrated that Model Parameterization is largely driven by theImplicit Baseline contrast, as Model Parameterization has a negligibleimpact on between-condition contrasts. Consistent with registeredhypotheses, the*Large Gain*versus*ImplicitBaseline*had the highest estimated median ICC. Contrary toregistered hypotheses, there was little evidence to suggest that analyticdecisions differentially impacted estimated median ICCs betweendevelopmental samples (e.g., oldest MLS/AHRB vs. younger ABCD data).Finally, the older samples (AHRB/MLS) had higher between-run andbetween-session estimated ICCs than the younger sample (ABCD).

### 3.4 Aim 1b: Effect of analytic decisions on Jaccard (binary) and Spearman (continuous) similarity estimates of group maps

Aim 1b proposed to evaluate the estimated group map similarity betweenmeasurement occasions (runs/sessions) using a Jaccard similarity for thresholdedbinary maps and a Spearman similarity for continuous measures across the 240pipeline permutations. The distribution of the estimates across the four modeloptions and three samples is reported inFigure5for Jaccard and suprathreshold Spearman similarity. Thespecification curve of Session 1 between-run estimates is reported inFigure 6for Spearman similarity (seeFig. S21for Jaccard).Based on the group-level Cohen’s*d*maps, there is a highsimilarity between the*Small Gain*and*LargeGain*versus*Implicit Baseline*(and*LargeGain*) contrasts that appear to be driven by the*ImplicitBaseline*condition and high similarity between Cue and Fixationmodels (seeFig.S22).

**Fig. 5.**
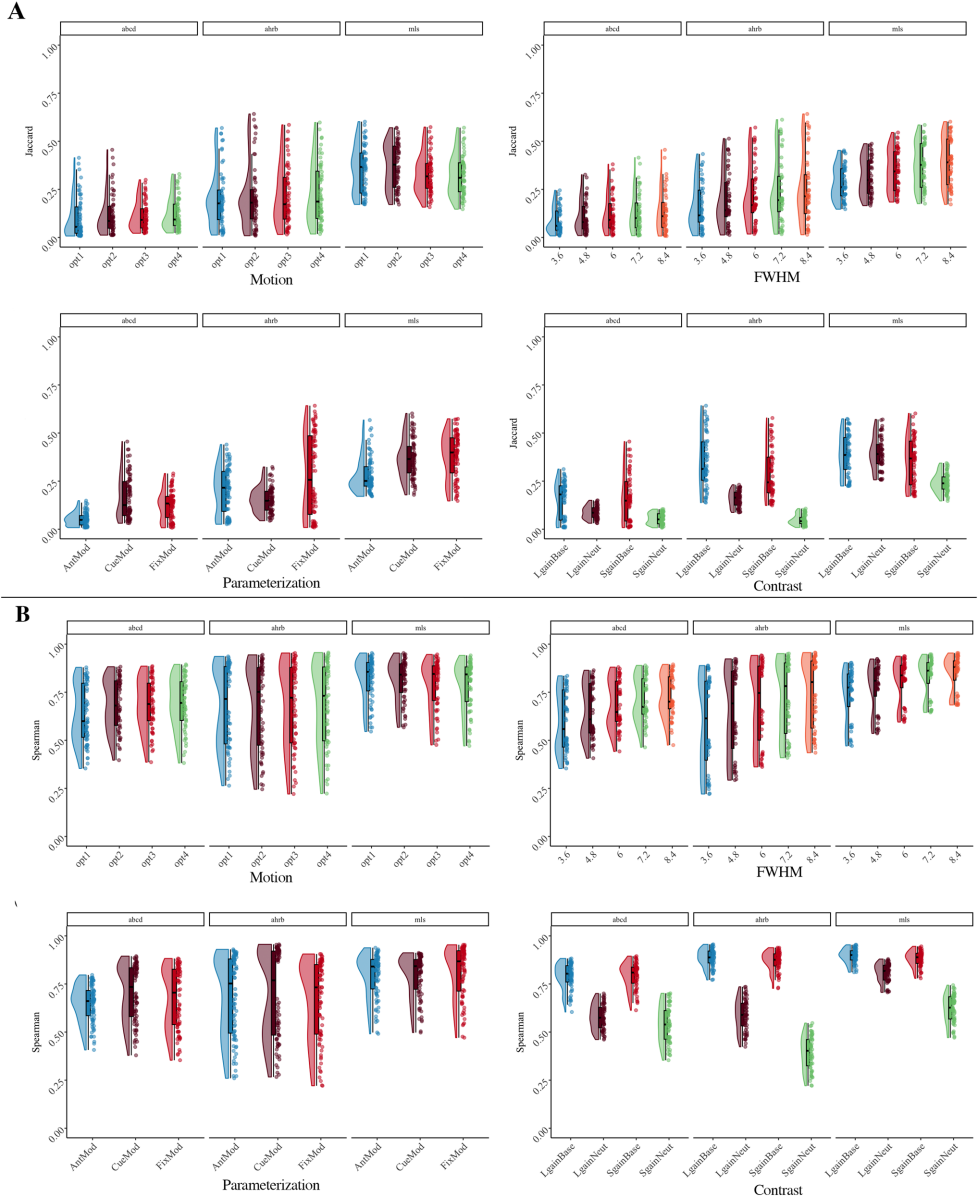
(A)*Jaccard*and (B)*suprathreshold SpearmanSession 1 Between-run*similarity estimates across [Four]analytic options for between-run reliability across the ABCD, AHRB, andMLS samples.

**Fig. 6.**
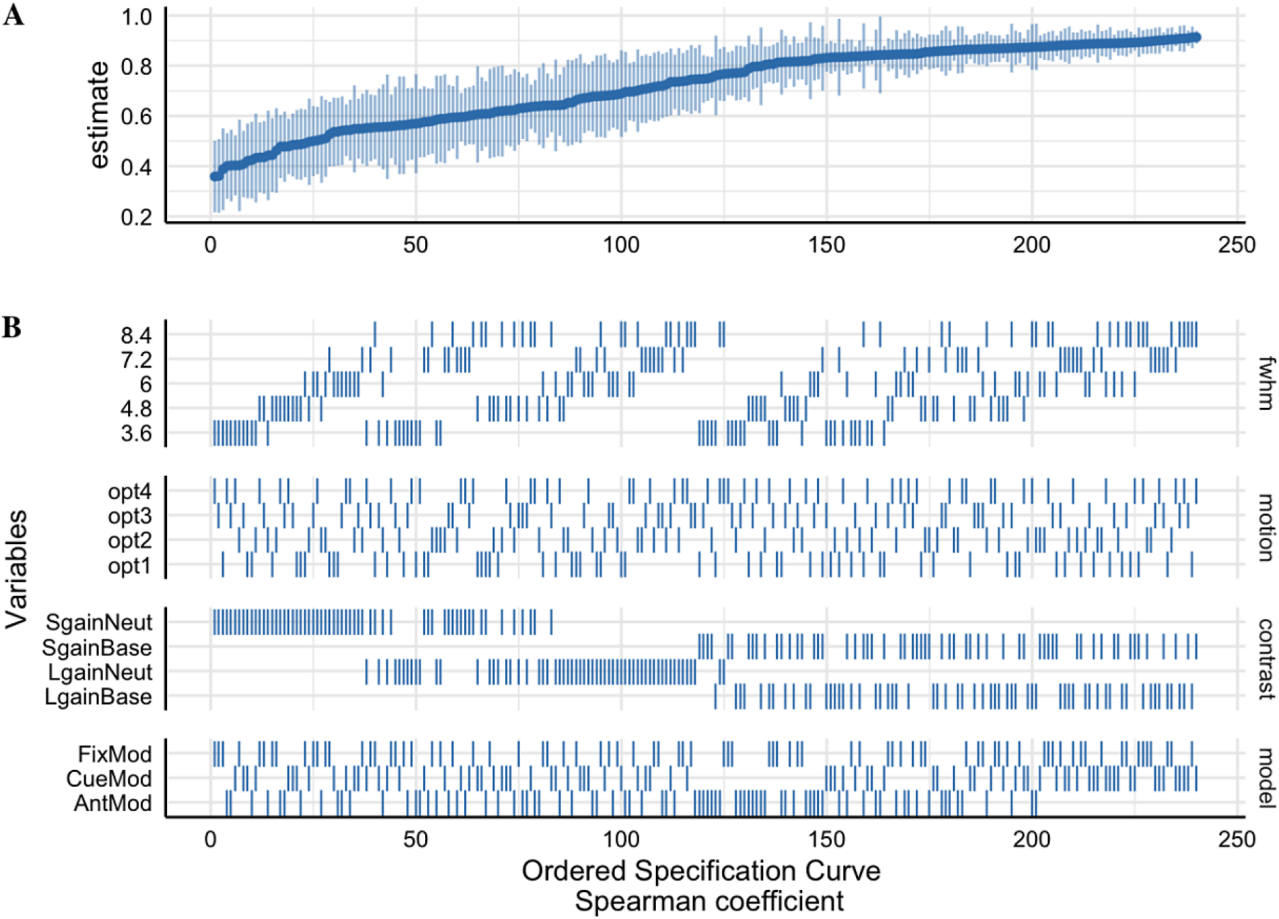
The suprathreshold Specification Curve of the*Session 1Between-run Spearman similarity*estimates across 240pipeline permutations for the ABCD, AHRB, and MLS samples. (A) Thedistribution of the point estimate (average) and distribution (errorbars) across the three samples. (B) The model options (four) associatedwith each estimate.

Similar to Aim 1a (TableS5;Fig. 2), on average theSession 1 between-run suprathreshold Spearman similarity is slightly lower thanthe suprathreshold between-session Spearman similarity (between-run: ABCD= .68 [range: .35 – .89]; AHRB = .73 [range: .22 –.96]; MLS = .84 [range: .47 – .96]; between-session: ABCD =.80 [range: .40 – .94]; AHRB = .82 [range: .32 – .97]; MLS= .87 [range: .59 – .97]). A similar trend is observed for theJaccard Similarity coefficient. The effects reported inFigure 5illustrate that the analytic categories have uniqueimpacts on the estimated Jaccard and suprathreshold Spearman coefficients. Whilethe Jaccard coefficient varies most across contrast and model parameterizationoptions (Fig. 5A), the Spearman similarityvaries most across FWHM and contrast type (Fig.5B). The specification curve for the Spearman similarity coefficientsillustrates a near ceiling similarity for estimates at the upper tail of theestimates and little variability across the three samples (Fig. 6). The HLM estimates indicate that a change from 3.6to 8.4 FWHM results in a*b = *.08 increase in Jaccardsimilarity and a*b = *.13 increase in Spearmansimilarity. Furthermore, the change from the contrast*LargeGain*versus*Implicit Baseline*to*LargeGain*versus*Neutral*results in a*b= *-.09 decrease in Jaccard Similarity and a*b= *-.20 decrease in Spearman similarity. While mostparameters are significant inTable 3,the effects vary in relative importance in the model. The variability in theestimated coefficients across 240 pipelines and 3 samples is best explained byContrast (marginal**∆**R^2^: .21) and modelparameterization (marginal**∆**R^2^: .05) for Jaccardsimilarity coefficient, and Contrast (marginal**∆**R^2^: .66) and FWHM (marginal**∆**R^2^: .08) for suprathreshold Spearmansimilarity coefficient. Surprisingly, the motion regressor options had anear-zero impact on the variability on both Jaccard and Spearman similaritycoefficients. Similar to Aim 1a,*post hoc*analyses illustratean interaction between Contrasts and Model Parameterization (Fig. S23), whereby thelargest driver of Model Parameterization differences in the Spearman*rho*similarity is as a function of the contrasts includedthe*Implicit Baseline*.

**Table 3.** Hierarchical linear model: (A) Linear associations between the analyticdecisions and the*Jaccard and Spearman suprathreshold*mask Session 1 between-run similarity and (B) the impact of the analyticcategory on the marginal R^2^.

**A. HLM Group-map Estimates**								
	Jaccard				Spearman			
*Predictors*	*b*	*CI*	*p*		*B*	*CI*		*p*
(Intercept)	.20	.09 – .31	<.001		.76	.69— .83		<.001
Reference [3.6]								
fwhm [4.8]	.03	.01 – .05	.004		.05	.04 – .07		<.001
fwhm [6.0]	.05	.03 – .07	<.001		.09	.07 – .10		<.001
fwhm [7.2]	.07	.05 – .09	<.001		.11	.10 – .13		<.001
fwhm [8.4]	.08	.06 – .10	<.001		.13	.12 – .15		<.001
Reference [opt1]								
motion [opt2]	.01	-.00 – .03	.13		.01	-.00 – .03		.05
motion [opt3]	.00	-.02 – .02	.85		.01	-.00 – .02		.20
motion [opt4]	.00	-.01 – .02	.69		.01	-.00 – .03		.08
Reference [AntMod]								
model [CueMod]	.05	.04 – .07	<.001		.02	.01 – .03		<.001
model [FixMod]	.08	.07 – .10	<.001		.01	-.00 – .02		.18
Reference [LgainBase]								
con [LgainNeut]	-.09	-.10 – -.07	<.001		-.20	-.21 – -.18		<.001
con [SgainBase]	-.03	-.05 – -.01	.001		-.01	-.02 – .00		.17
con [SgainNeut]	-.18	-.20 – -.16	<.001		-.34	-.35 – -.32		<.001

**B. Analytic Category Model Impact**								
**Comparison**	χ ^2^	Orig R ^2^	New R ^2^	∆R ^2^	χ ^2^	Orig R ^2^	New R ^2^	∆R ^2^
[Full] vs. [New – fwhm]	78	.30	.26	.04	292	.74	.66	.08
[Full] vs. [New – motion]	3	.30	.30	.00	5	.74	.74	.00
[Full] vs. [New – model]	104	.30	.25	.05	14	.74	.73	.01
[Full] vs. [New – con]	348	.30	.09	.21	1,205	.74	.08	.66

The group-level maps indicate a notable difference in contrasts usingthe*Neutral*and*Implicit Baseline*conditions (NeuroVault ABCD:https://identifiers.org/neurovault.collection:17171AHRB:https://identifiers.org/neurovault.collection:16605; MLS:https://identifiers.org/neurovault.collection:16606). AsFigureS22shows, the*Large Gain*versus*Neutral*contrast reflects a qualitativelycomparable activation map across Cue, Fixation, and AnticipationModels. On the other hand, the*Large Gain*versus*Implicit Baseline*contrast differs acrossmodels, where the most notable pattern is that the Cue model isnegative of the Fixation model across the samples. Specifically, inABCD, AHRB, and MLS, there is increased negative activity in theinsular, visual, motor, and visual areas, in the Cue Model, and thispattern is mostly opposite of the Fixation Model. Meanwhile, in theAnticipation model, there is high positive activity in the dorsalstriatal, SMA, and Insular regions. This reflects the variablemeanings of*Implicit Baseline*across the models.The relative symmetry between the Cue and Fixation models isconsistent with the fact that each serves as the$B_{0}$in the models, for example,$B_{1\;\left[Condition\;A,\;Cue\right]}-\;B_{0\left[All\;Fixation\;\;+\;\;Probe\;Phase\right]\;}$and$B_{1\left[Condition\;A,\;fixation\right]}-\;B_{0\left[All\;Cue\;\;+\;\;Probe\;Phase\right]\;}$.The Anticipation model is more variable as it is contrasted with amore narrow phase of the task, for example,$B_{1\left[Condition\;A,\;Cue+Fixation\right]}-\;B_{0\left[Probe\;Phase\right]\;}$.

#### 3.4.1 Summary of findings for Aim 1b

Similar to Aim 1a, on average, the suprathreshold Session 1 between-runSpearman and Jaccard similarity is slightly lower between-sessionsimilarity. Spearman similarity meaningfully differed across Contrast, ModelParametrization, and Smoothing, and it is near the ceiling for the uppertail of the Spearman similarity estimates. Like Aim 1a, ModelParametrization is driven by the Implicit Baseline. Finally, mean-basedgroup activity maps illustrate that the Cue and Fixation models are oppositeof each other when the contrast is a between-condition and implicit baselinecomparison.

### 3.5 Aim 2: Effect of analytic decisions on median BS/WS estimates from individual continuous maps

Aim 2 proposed to evaluate the changes in the between-subject variance (BS) andwithin-subject variance (WS) components that differentially relate to theICC(3,1) across the 240 workflow permutations. The supra- and subthresholddistributions across the four model options and three samples are reported inSupplemental Figures S24and S25and specification curves for BS inSupplemental Figure S28and WS inSupplementalFigure S29. The HLM estimates (Table3) suggest that the Implicit Baseline contrasts increase BS varianceand more stringent motion correction decreases BS variance, and ImplicitBaseline contrasts and larger smoothing kernels reduce WS variance. Thevariability in the estimated BS coefficients across 240 pipelines and 3 samplesis best explained by Contrast (**∆**R^2^: .30), modelparameterization (**∆**R^2^: .10), and then motion(**∆**R^2^: .04). The variability in the estimatedWS coefficients across 240 pipelines and 3 samples is best explained by FWHM(**∆**R^2^: .21), Contrast(**∆**R^2^: .14), and then model parameterization(**∆**R^2^: .10). A comparable trend is observed inthe between-session estimates (Table S9), with the exception of Contrast selectionexplaining more variability (**∆**R^2^: .26) than FWHM(**∆**R^2^: .16). We avoid interpreting thesubthreshold mask as it includes regions that are high-noise (e.g., white matterand ventricles) and drop-out areas (e.g., cerebellar and medial orbital frontalcortex) which exaggerates the BS and WS components.

### 3.6 Aim 3: Stability of the ICC, BS, and WS components across sample size

**Fig. 7.**
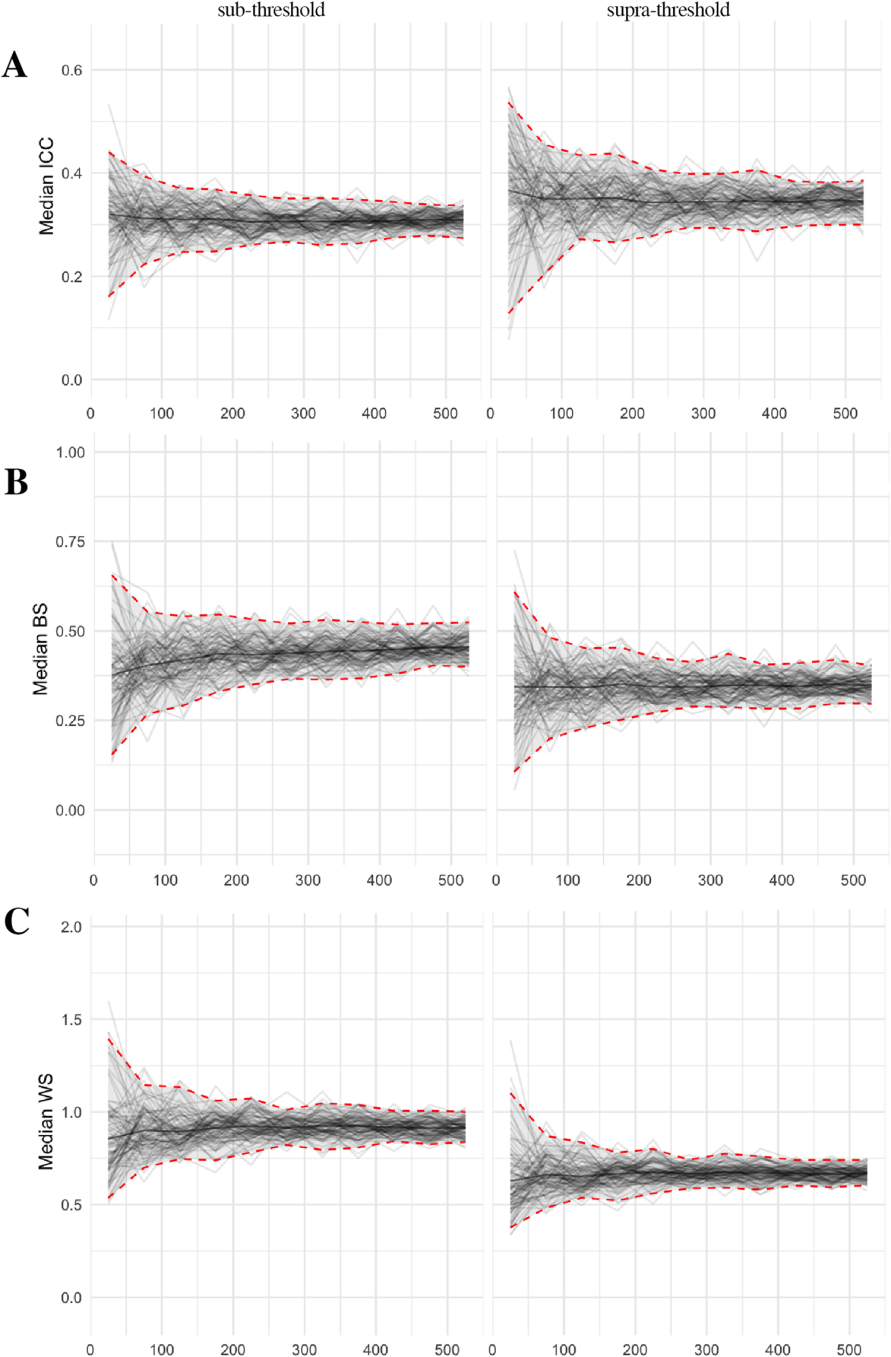
Changes in the supra- and subthreshold median (A) intraclass correlation(ICC), (B) between-subject variance (BS), and (C) within-subjectvariance (WS) estimate in the ABCD sample for*N*25 to525 with 100 bootstraps at each*N*. Note: Based on thetop model fromFigure 2:*Small Gain*versus*ImplicitBaseline*Contrast, “CueMod” Model, Motionoption 1 and FWHM 8.4.

As expected, based on sampling theory which demonstrates that variabilitydecreases as a function of the square root of*N*, thevariability in estimates decreased as*N*increased.Specifically, the bootstrapped estimates for the median ICC, BS, and WS changeslowly at higher intervals of*N*(Fig. 7). In*post hoc*comparisons of whole brainvoxel-wise ICC maps, the largest variability occurs below N = 275. Asreported inSupplementalFigure S36, at*N = *25, the minimum andmaximum median whole brain ICC maps have a wider voxel-wise distribution of ICCvalues which are notably different (Cohen’s*d*=1.9). With increasing*N*, Cohen’s*d*ofthe whole brain voxel-wise distributions between the minimum and maximum 3D ICCmaps narrows,*d*= 1.4 at*N*= 225and*d*= 1.0 at*N*= 525,respectively.

### 3.7 Post hoc analyses

An exploratory set of analyses was performed to evaluate (1) the effect ofanalytic decisions on ICC for the Left and Right Nucleus Accumbens and (2) theassociation between voxel-wise Cohen’s*d*estimates atthe group-level and the voxel-wise ICC maps. These are reported inSupplemental Section2.6.

## 4 Discussion

Understanding the analytic decisions that may consistently increase individual-and/or group-level reliability estimates has implications for the study ofindividual differences using fMRI. The current study expands on previous work bysimultaneously evaluating the effects of smoothing, motion correction, taskparameterization, and contrast selection on the continuous and binary reliabilityestimates of BOLD activity during the MID task for run- and session-level dataacross three independent samples. The five major findings are: (1) The ICC(3,1)test–retest reliability estimates in the MID task are consistently low; (2)group-level estimates of reliability are higher than individual [ICC] estimates; (3)Contrast selection and Model Parameterization have the largest impact on median ICCestimates, and Smoothing and Contrast selection have the largest impact onsimilarity estimates; however, gains in reliability across different contrasts comeat the cost of interpretability and may differ; (4) Motion correction strategies inthese analyses did not meaningfully improve individual or group similarity estimatesand, in some cases,*reduced*estimates of reliability; and (5) themedian ICC estimate varied across sample size but the variability decreased withincreased sample size. Excluding some differences, the results are relativelyconsistent across the three samples, runs and sessions, providing a comprehensiveoverview of how analytic decisions at the GLM impact reliability of estimated BOLDin commonly used versions of the MID task.

The results from these multiverse analyses align with earlier studies, demonstratingthat ICC estimates for univariate task-fMRI are relatively low, making theminsufficient for individual differences research (Elliott et al., 2020;Kennedy et al.,2022). Consistent withElliott et al(2020), reliability estimates in the subthreshold (or nontarget mask) arelower than those in the suprathreshold of the MID task (target mask). The range ofmedian ICCs varied across analytic decisions. Using commonly employed cutoffs (Cicchetti & Sparrow, 1981;Elliott et al., 2020;Noble et al., 2019), ICC estimates for*LargeGain*versus*Neutral*contrast are in the“Poor” range and the*Large Gain*versus*Implicit Baseline*contrast ranged between “Poor”and “Fair” across the three samples. Test–retest reliabilityfor the*Large Gain*(*Small Gain*) versus*Implicit Baseline*contrast is modulated by ModelParameterization, whereby the Cue Model had a meaningfully higher reliability thanthe Anticipation Model. However, this may come at the cost of validity, which isdiscussed below. Nevertheless, based on voxel-wise distributions from the topperforming model (Model: Cue Model, Contrast:*Small Gain*versus*Implicit Baseline*, Motion Correction: None, Smoothing: 8.4 mmkernel), visual and motor regions had the highest ICCs, in the “Fair”to “Good” range.*Post hoc*analyses of the bilateralNAc illustrate that, on average, ICC estimates in this region of interest are in the“Poor” range. Notably, ICCs in this*post hoc*regionwere not meaningfully impacted by Model Parameterization but were impacted byContrast and Motion correction, suggesting that test–retest reliability maybe uniquely impacted by analytic strategy depending on the voxels underconsideration. These findings illustrate that the test–retest reliability ofthe MID task is relatively low, even in the most common ROI such as the Left andRight NAc. WhileKennedy et al. (2022, p. 13)speculated that low reliabilities in the ABCD sample may be attributed to theparticipants’ young age, our results demonstrate that median ICC estimatesare*higher*in older than in younger samples, but reliabilityestimates in the MID task remain consistently low across early adolescents and lateadolescents/young adults. To understand how analytic strategies differentiallyimpact ICCs in different brain regions, we encourage future researchers to use thepublicly available estimated maps to probe this question further.

Consistent withFröhner et al. (2019),the group-level maps are not always representative of the individual-level mapsacross analytic decisions. On average, the Spearman*rho*, Jaccardcoefficients, and median ICC estimates are higher for the between-session than forthe between-run estimates. Consistently, Spearman*rho*estimates aremeaningfully higher for suprathreshold group maps than for suprathreshold median ICCestimates derived from individual maps. This suggests that across each of the threesamples, the MID task is relatively effective at eliciting a group-level activationmap; however, the individual estimates are lower and more variable. In the contextof the MID task, the between-run and between-session effects may be the result ofwithin-session effects*decreasing*across runs (Demidenko, Mumford, et al., 2024). Notably, the higherbetween-session than between-run reliabilities are inconsistent with values reportedin previous work (Fröhner et al.,2019), this is likely the result of those between-run estimates being basedon randomly split-half (within runs) which are inflated as a result of dependenciesin the model estimates within runs (Mumford et al.,2014). Nevertheless, the results here emphasize that group-level maps andgroup similarity are not a good indicator of individual-level reliabilities. This isunsurprising, considering that the MID task design was optimized to elicit activityin anatomical regions at a group level and for averaged time courses within ananatomical region (Knutson et al., 2003).

A major question of these analyses was: Are there decisions that*consistently*result in higher individual- (continuous) and/orgroup-level reliability estimates (continuous/binary)? The results across theanalytic choices illustrate that reliability estimates are impacted most bycontrast, model parameterization, and smoothing decisions. Across the three samples,for between-run and between-session estimates, the contrast type had the largestinfluence of individual and group reliability estimates. Consistent with previousreports (Baranger et al., 2021;Han et al., 2022;Kennedy et al., 2022;Vetter et al., 2015,^2017^), thecontrast*Large Gain*(and*Small Gain*) versus*Implicit Baseline*had meaningfully higher estimated ICC,Jaccard, and Spearman*rho*similarity estimates than the*Large Gain*versus*Neutral*contrast. Theestimated ICC and Spearman*rho*coefficients for contrasts aremodulated by the model parameterization, whereby the conditions including the*Implicit Baseline*are highest for the Cue Modelparameterization. Conversely, ICC and similarity estimates are relatively stableacross the three model parameterizations when comparisons are against the*Neutral*condition. Whether using contrasts or percent signalchanges, estimates of BOLD activity suffer from decreases in reliability due todifference scores (Hedge et al., 2018). Wheregains are observed from the less reliable*Large Gain*versus*Neutral*to the more reliable*Large Gain*versus*Implicit Baseline*contrast, it comes at the cost ofinterpretability and face validity that is expected in the estimated BOLD activity.Finally, higher FWHM smoothing kernels positively impacted between-run andbetween-session median ICC estimates and Spearman*rho*similarityestimates, whereas motion correction strategies had a smaller but negative impact onthese estimates (i.e., more stringent motion correction reduced reliabilityestimates). Decisions to smooth in the MID task are especially important given thatlarger smoothing kernels have been reported to spatially bias reward-relatedactivity in the MID task (Sacchet & Knutson,2013). In general, variability in reliability estimates decreased withlarge sample sizes.

Improvements in estimated reliability as a function of contrast selection may come atthe cost of interpretability. For example, in the context of the*LargeGain*versus*Neutral*contrast, despite differences inthe estimated efficiencies, the ICC estimates are relatively stable across the modelparameterizations in each of the three samples, and the activation patterns areinterpretable at the group level. In the context of the*Large Gain*versus*Implicit Baseline*contrast, there are meaningful differencesin the ICC estimates across model parameterizations, whereby the Cue and Fixationmodels demonstrate a substantial improvement over the Anticipation modelparameterization, but the group-level activity patterns are less interpretable. As aresearcher looking for BOLD estimates that are consistent from run-to-run orsession-to-session for individual participants, the*ImplicitBaseline*suggests a considerable and valuable improvement on thereliability of estimated values. However, the difference of means for the*Implicit Baseline*is complicated by the intercept in the GLM atthe first level. For example, in the Cue Model parameterization, the intercept takeson the average for the unmodeled phase of the task which includes the fixation cross(between cue and probe phase) and the probe response phase. In this instance,isolating the difference of [Cue*Large Gain*] – [Fixation+ Probe phase] to a specific cognitive function becomes especiallychallenging (Poldrack & Yarkoni, 2016;Price & Friston, 1997). It is wellrecognized that different definitions of “baseline,” whether rest,passive, or task-related, in task-fMRI will result in different activation patterns(Newman et al., 2001). The use of“neutral” or “fixation” is a cause for caution as itimpacts interpretability in various fMRI task designs (Balodis & Potenza, 2015;Filkowski & Haas, 2017). Here, we illustrated howcontrasts with the unmodeled phases of a task (*Implicit Baseline*)may improve reliability estimates but may be heavily biased by the activity patternsthroughout the task and diminish the validity of the measure. It is reasonable tosuspect that subtle modeling deviations between similar and different task designswould further complicate comparisons between studies when using an*ImplicitBaseline*condition.

In the context of test–retest reliability of estimated BOLD activity, it isimportant to consider alternative methods to improve reliability, estimationprocedures, and considerations of what a “reliable” BOLD estimateimplies. In general, the evidence here illustrates that the test–retestreliability for the modified version of the MID task is consistently low using theintraclass correlation (ICC[3,1]), even at its maximum. The analytic decisions atthe GLM modeling phase demonstrated improvements in reliability from between run tobetween session. Higher between-session reliability may be related to decreasingactivity from early to later runs (Demidenko,Mumford, et al., 2024) or based on the sessions being an average of tworuns/increased trials (Han et al., 2022;Ooi et al., 2024). In the currentanalyses, we focused on univariate maps and the parametric, voxel-wise ICCestimation procedures (ICC[3,1]). Parametric and nonparametric multivariate methodsare reported to improve reliability estimates over univariate estimates usingmultidimensional BOLD data (Gell et al.,2023;Noble et al., 2021). Forexample, I2C2 is a parametric method that pools variance across images to estimate aglobal estimate of reliability using a comparable ratio as ICC (Shou et al., 2013) and the discriminability statistic isa nonparametric statistic that is a global index of reliability testing whether thebetween-subject distance between voxels is greater than the within-subject voxels(Bridgeford et al., 2021). Each of thesemetrics uniquely summarizes the within- and between-subject variability of theestimated BOLD data and so a consensus and definition of reliability in task-fMRIremain a challenge (Bennett & Miller,2010). In our analyses, we used the ICC as it estimated the reliabilityfor each voxel in an easy-to-interpret coefficient that is useful in commonbrain-behavior studies. Cutoffs from the self-report literature (Cicchetti & Sparrow, 1981) are often leveraged infMRI research (Elliott et al., 2020;Noble et al., 2019); however, these cutoffsshould depend on the optimal level of precision necessary for the question andreasonable for the methods (Bennett & Miller,2010;Lance et al., 2006). Somerecommendations have been made to use bias corrections in developmental samples toadjust for suboptimal levels of reliability (Hertinget al., 2017), but these corrections should be used cautiously as they donot account for the underlying problems of the measure or the complexities in thedata that prevent accurate measurement of the latent process (Nunnally, 1978).

## 5 Study Considerations

The analytic decisions in the current analyses focused primarily on a subset ofdecisions at the First Level GLM model and its impact on estimates andsupra-/subthreshold masks. As a result, other decisions were not considered that mayarise at the preprocessing (Li et al., 2021),assumed hemodynamic response function (Kao et al.,2013;Lindquist et al., 2009),cardiac and respiratory correction (Allen et al.,2023;Birn et al., 2006), and theeffects of different methods of signal distortion correction (Montez et al., 2023). Furthermore, we focused onvoxel-wise estimates of reliability which are typically noisier than*apriori*anatomical regions. It is unclear how much interpretation wouldchange if ICC estimates were compared across variable parcellations. Nevertheless,we shared all aggregate maps for the three samples and the preprocessed data for theMLS/AHRB samples to facilitate reanalysis.

The results provide a comprehensive overview of individual and group reliabilityestimates for the modified version of the MID task, but it is challenging to inferhow reflective these results are of alternate MID designs and different rewardtasks. Based on prior reports of low test–retest reliabilities in task fMR,if a sufficient sample size is used, we suspect that results may be comparable withother MID and reward task designs. Future research should consider how reliabilityestimates change as a function of modeling decisions in different taskparadigms.

## 6 Conclusion

With the increasing interest in test–retest reliability in task fMRI andmethods for improving reliability estimates of BOLD, the current study evaluatedwhich decisions at the GLM model improved group and individual reliability estimatesof reliability. In general, the findings illustrate that the MID task groupactivation maps are more reliable than individual maps across testing occasions andindependent samples. Across group and individual models, between-session estimatesare consistently higher than between-run estimates of reliability. Furthermore,estimates of reliability were more variable at the median fMRI sample size andstabilized with*N*. While individual estimates of reliability arelow (ICC[3,1]), Contrasts and Model Parameterization meaningfully improvedtest–retest reliability. However, the improvement in reliability came at thecost of interpretability and may be region specific in the current version of theMID task. This underscores the importance of evaluating reliability in largersamples sizes and ensuring improved estimates reflect the neural processes ofinterest. While Model Parameterization and Contrast selection had the largest impacton voxel-wise ICCs, further work is needed to expand on these findings by evaluatingalternative brain regions and analytic decisions that may result in improvedtest–retest reliability that may be meaningful in individual differencesresearch.

## Supplementary Material

Supplementary Material

## Data Availability

*Adolescent Brain Cognitive Development*(ABCD) data: The ABCD BIDSdata, MRIQC v23.1.0 and fMRIPrep v23.1.4 derivatives can be accessed through theABCD-BIDS Community Collection (ABCC) with an established Data Use Agreement (seehttps://abcdstudy.org/). The dataused in these analyses will be available at a future release onto the NationalInstitute of Mental Health Data Archive. The complete set of group-level and ICCmaps is publicly available on Neurovault for ABCD (6,180 images;https://identifiers.org/neurovault.collection:17171). *Michigan Longitudinal Study*(MLS) and*Adolescent Health RiskBehavior*(AHRB) data: The BIDS inputs, fMRIPrep v23.1.4 and MRIQCv23.1.0 derivates are available on OpenNeuro.org (MLS:https://doi.org/10.18112/openneuro.ds005027.v1.0.1AHRB:https://doi.org/10.18112/openneuro.ds005012.v1.0.1). The complete set ofgroup-level and ICC maps is publicly available on Neurovault for MLS (2,400 images;https://identifiers.org/neurovault.collection:16606) and AHRB (2,400images;https://identifiers.org/neurovault.collection:16605). *R and Python code*: The*.html*and*.rmd*file containing the code to be run on extracted estimatesfrom reliability maps are available on Github with the associated output filescontaining the estimates across the models and samples. Likewise, all of the codefor first level, fixed effect, group, and ICC models are available online athttps://github.com/demidenm/Multiverse_Reliabilityand DOI:10.5281/zenodo.12701228x.

## Footnotes

* Reliability of parameter estimates at the individual level and thresholdedactivation maps at the group level have previously been distinguished as“reliability” and “reproducibility” of BOLDactivity, respectively (Bennett & Miller,2013;Plichta et al., 2012;Zuo et al., 2014). We elect to referto individual and group estimates as distinct forms of reliability and use“reproducibility” to refer to a broader set of concepts describingvarious aspects of the ability to reproduce or generalize a research finding(e.g.,Goodman et al., 2016).

† For the Stage 1 submission, the data for the different studies were not fullyaccessed, inspected, preprocessed, or analyzed. Thus, the sample sizeapproximations. Thus, the sample size approximations. The final*N*for each sample is expected to deviate from theapproximated values because of complete data availability and quality controlexclusions. See theresultssection foranalytic*N*s in Stage 2.

‡ At Stage 1, the sample was based on an approximation. During Stage 2, we realizedit would be more effective to take advantage of the complete available data byusing standardized effect Cohen’s*d*maps.
